# Phosphorylation of the RSRSP stretch is critical for splicing regulation by RNA-Binding Motif Protein 20 (RBM20) through nuclear localization

**DOI:** 10.1038/s41598-018-26624-w

**Published:** 2018-06-12

**Authors:** Rie Murayama, Mariko Kimura-Asami, Marina Togo-Ohno, Yumiko Yamasaki-Kato, Taeko K. Naruse, Takeshi Yamamoto, Takeharu Hayashi, Tomohiko Ai, Katherine G. Spoonamore, Richard J. Kovacs, Matteo Vatta, Mai Iizuka, Masumi Saito, Shotaro Wani, Yuichi Hiraoka, Akinori Kimura, Hidehito Kuroyanagi

**Affiliations:** 1 Laboratory of Gene Expression, Medical Research Institute, Tokyo Medical and Dental University (TMDU), Bunkyo-ku, Tokyo 113-8510 Japan; 2 Graduate School of Biomedical Science, Tokyo Medical and Dental University (TMDU), Bunkyo-ku, Tokyo 113-8510 Japan; 3 Laboratory for Integrated Research Projects on Intractable Diseases Advanced Technology Laboratories, Medical Research Institute, Tokyo Medical and Dental University (TMDU), Bunkyo-ku, Tokyo 113-8510 Japan; 4 Department of Molecular Pathogenesis, Medical Research Institute, Tokyo Medical and Dental University (TMDU), Bunkyo-ku, Tokyo 113-8510 Japan; 5 Department of Medicine and Clinical Science, Division of Cardiology, Yamaguchi University Graduate School of Medicine, Ube, Yamaguchi 755-8505 Japan; 6 Department of Medicine, Krannert Institute of Cardiology, Indiana University School of Medicine, Indianapolis, IN 46202 USA; 7 Department of Medical and Molecular Genetics, Indiana University School of Medicine, Indianapolis, IN 46202 USA; 8 Laboratory of Molecular Neuroscience, Medical Research Institute, Tokyo Medical and Dental University (TMDU), Bunkyo-ku, Tokyo 113-8510 Japan; 9 Laboratory of Recombinant Animals, Medical Research Institute, Tokyo Medical and Dental University (TMDU), Bunkyo-ku, Tokyo 113-8510 Japan; 10 Department of Microbiology, Immunology and Molecular Genetics, University of California, Los Angeles (UCLA), Los Angeles, CA USA

## Abstract

RBM20 is a major regulator of heart-specific alternative pre-mRNA splicing of *TTN* encoding a giant sarcomeric protein titin. Mutation in *RBM20* is linked to autosomal-dominant familial dilated cardiomyopathy (DCM), yet most of the *RBM20* missense mutations in familial and sporadic cases were mapped to an RSRSP stretch in an arginine/serine-rich region of which function remains unknown. In the present study, we identified an R634W missense mutation within the stretch and a G1031X nonsense mutation in cohorts of DCM patients. We demonstrate that the two serine residues in the RSRSP stretch are constitutively phosphorylated and mutations in the stretch disturb nuclear localization of RBM20. *Rbm20*^*S637A*^ knock-in mouse mimicking an S635A mutation reported in a familial case showed a remarkable effect on titin isoform expression like in a patient carrying the mutation. These results revealed the function of the RSRSP stretch as a critical part of a nuclear localization signal and offer the *Rbm20*^*S637A*^ mouse as a good model for *in vivo* study.

## Introduction

Dilated cardiomyopathy (DCM) is a heart disease characterized by left ventricular dilatation and systolic dysfunction^1,2^. Within a group idiopathic DCM, 30-50% are familial, with autosomal dominant inheritance in most cases^2,3^. Today, next-generation sequencing methods have identified over 400 potentially causative mutations in nearly 60 genes of various functional groups including muscle contraction, Ca^2+^ handling and nuclear functions both in familial and sporadic DCM cases^4^. Such complexity in molecular genetics of DCM, however, makes it challenging to elucidate mechanisms eventually leading to the common phenotypes of DCM^1^.

*RBM20*, encoding RNA binding motif protein 20 (RBM20), was identified as one of the genes linked to autosomal-dominant familial DCM^5^. RBM20 has later been identified as an alternative splicing regulator of a gene responsible for expression of an aberrantly giant isoform of a sarcomeric protein titin in a spontaneously occurring rat strain^6^. Titin is the largest known protein and the gene for titin, *TTN*, has the largest number of exons (363 in humans) in vertebrates^7–9^. One titin molecule spans from the Z-disk through the M-band of a sarcomere, a contractile unit of striated muscles, and functions as a molecular spring contributing to passive tension of cardiomyocytes^10–12^. In adult hearts, two major titin isoforms are expressed: N2B is a shorter isoform where exon 50 is spliced to exon 219 and exons in between are all skipped; N2BA is a mixture of multiple intermediate isoforms where exons 102–108 encoding N2A-specific element and some of variable exons 51 through 100 encoding immunoglobulin (Ig) repeats are included^13^. In an *Rbm20* mutant rat strain lacking nearly all of the *Rbm20* exons, titin N2B is no longer expressed and N2BA is predominant in the heterozygotes, and an aberrantly giant isoform N2BA-G, in which all of the exons 51–218 are included, is exclusively expressed in the homozygotes, indicating that RBM20 is a key regulator of *Ttn* pre-mRNA processing in the adult heart^6^. Since titin-based passive tension is negatively correlated with its molecular size^10,12,14^, the ratio of the isoforms and the amount of the titin proteins are considered to impact passive stiffness of the myocardium and progression of DCM^15–17^.

RBM20 protein consists of multiple evolutionarily conserved domains including one RNA-recognition motif (RRM) domain and two zinc finger (ZnF) domains^6^, and directly binds to its target pre-mRNAs in cardiomyocytes^18^. Nevertheless, extensive search for mutations in *RBM20* in familial and sporadic DCM patients revealed a hotspot of missense mutations at a highly conserved RSRSP stretch within an arginine/serine (RS)-rich region and not in the putative RNA-binding domains^5,6,19–22^. Some missense and nonsense mutations outside of the hotspot were also identified in such screenings^19,20,23,24^, yet neither family study nor functional study was performed and significance of such mutations remains to be elucidated. This situation is unusual considering that most of missense mutations were mapped to the RRM domains in our previous genetic screenings for loss- or reduction-of-function mutants for splicing factors^25–27^. It therefore remains to be unraveled why any residue in the RSRSP stretch is critical for the function of RBM20 and to what extent the mutations outside of the stretch affect the function.

In this study, we identified one missense mutation R634W in the RSRSP stretch and one truncation mutation G1031X losing one of the ZnF domains in *RBM20* in DCM patients. We utilized a fluorescence splicing reporter to evaluate the function of RBM20 and demonstrate that phosphorylation of the RSRSP stretch is critical for nuclear localization of the splicing regulator. We also investigated functional alterations caused by the DCM-associated *RBM20* mutations including three other missense variants that were reported to be found in other DCM patients.

## Materials and Methods

### Identification of RBM20 mutations in DCM patients

*RBM20* from DCM patients were screened for mutations in exons and exon-intron boundaries by using next-generation sequencing or by Sanger sequencing with di-deoxy chain termination method and automated nucleotide sequencers. Identified mutations were confirmed by Sanger sequencing in the proband patients and their family relatives by Sanger sequencing. Sequences of primers used in this study will be published elsewhere and are available upon request. Written informed consent for genetic analysis was obtained from patients when the patients were 16 years of age or older and/or from legal guardians such as their parents when the patients were under 16 years of age, as well as from family members who were 16 years of age or older. Unaffected family members under 16 years of age were not included in the genetic testing study. All research was performed in accordance with the guidelines for human genome analysis in Japan and USA, and the study protocol was approved by the Ethics Committee of the Medical Research Institute, Tokyo Medical and Dental University (TMDU) and that of the Indiana University School of Medicine.

### *In silico* prediction of RBM20 missense mutations

*In silico* analysis of missense variants was performed using Polyphen-2, SIFT, Mutation Taster, PROVEAN and FATHMM^28–32^. The variants were classified “Damaging” if they were determined to be “probably damaging” or “possibly damaging” in Polyphen-2, “damaging” in SIFT, “deleterious” in PROVEAN, “damaging” in FATHMM or “disease causing” in Mutation Taster. The variants were also evaluated by ClinVar database^33^. The variants were classified in accordance with the American College of Medical Genetics and Genomics (ACMG) Standards and Guidelines^34^.

### Plasmid construction

The *Ttn* splicing reporter minigene was constructed by using Gateway technology (Invitrogen) as follows. Genomic DNA fragments of *Ttn* spanning from exon 50 through exon 51 and from exon 218 through exon 219 were amplified by using PrimeSTAR GXL DNA Polymerase (Takara) and cloned into Gateway pENTR-L1/R5 vector (Invitrogen). A human histone H2B fragment was amplified from HeLa genomic DNA and cloned into a pDEST-cDNA3 vector backbone^25^ by using In-Fusion HD (Takara), and a cryptic splice site was mutagenized for constructing pDEST-cDNA3-H2B(RMver). EGFP cDNA with a 6x (Gly-Gly-Ser) linker^35^, porcine teschovirus-1 2 A fragment and mCherry cDNA (Clontech) were assembled in Gateway pENTR-L5/L2 vector (Invitrogen). Finally, the *TtnE50-E51E218-E219-EGFP/mCherry* minigene was constructed by assembling the Entry vectors and pDEST-cDNA3-H2B(RMver) with LR clonase II Plus (Invitrogen). The primers used to amplify the DNA fragments are available in Supplementary Table 1.

A full-length RBM20 cDNA was amplified from mouse heart cDNAs by using PrimeSTAR GXL (Takara) and cloned into pENTR/D-TOPO (Invitrogen). Entry vectors for RBM20 point mutants and deletion mutants were constructed by using PCR-based techniques with PrimeSTAR GXL (Takara). Expression vectors for N-terminally FLAG-tagged wild-type and mutant RBM20 proteins were generated by homologous recombination between pDEST-cDNA3-FLAG or pDEST-cDNA3-FLAG-3xNLS and the Entry vectors with LR clonase II (Invitrogen). pDEST-cDNA3-FLAG was constructed by converting pcDNA3-FLAG into a Destination vector^36^ and pDEST-cDNA3-FLAG-3xNLS was constructed by inserting three copies of SV40 nuclear localization signals (NLSs) into pDEST-cDNA3-FLAG. Wild-type and mutant RBM20(517–657) cDNA fragments were amplified from RBM20 expression vectors and cloned into a pcDNA3-FLAG vector backbone with In-Fusion HD (Takara). Sequences of primers used in the construction are listed in Supplementary Table 1 and sequence information of all the vectors is available upon request.

### Cell culture and transfection

HeLa and HEK293T cells were cultured in DMEM Medium (Nacalai) supplemented with 10% fetal bovine serum at 37 °C with 5% CO_2_. Plasmid DNAs were transfected by using FuGENE HD (Promega) according to the manufacturer’s instruction. The *Ttn* reporter minigene and the expression vectors for FLAG-tagged wild-type or mutant RBM20 proteins were co-transfected in a 1:4 mixture. Fluorescence images of fluorescent proteins were acquired 24–36 hours after transfection by using Leica system, and then, the cells were harvested for total RNA preparation and RT-PCR analysis

### Immunofluorescence staining and microphotography

HeLa cells transfected with FLAG-tagged RBM20 expression vectors were fixed with 1–4% paraformaldehyde in PBS for 10 min. The cells were permeabilized with PBS containing 0.1% Triton X-100 and 2% normal goat serum for 30 min and were stained with 2 µg/ml anti-FLAG monoclonal antibody (M2, Sigma-Aldrich) for 1 hour, 2 µg/ml Alexa546-conjugated goat anti-mouse IgG (Molecular Probes) for 30 min and DAPI (Invitrogen). Fluorescence images were captured by using a compound microscope (DM6000B, Leica) equipped with a color, cooled CCD camera (DFC310FX, Leica) and processed by using LAS AF (Leica) and Photoshop CC (Adobe).

### Total RNA extraction and RT-PCR

Total RNAs from HeLa and HEK293T cells were extracted by using Sepasol-RNA I Super G (Nacalai), treated with RQ1 RNase-free DNase (Promega) and reverse transcribed with PrimeScript II and oligo dT (Takara). Total RNAs from mouse heart were extracted by using RNeasy Plus Mini kit with DNase I (Qiagen) and reverse transcribed with PrimeScript II, random hexamers and oligo dT (Takara). Semi-quantitative PCRs were performed by using PrimeStarGXL or ExTaq (Takara) and the PCR products were analyzed by utilizing Bioanalyzer 2100 Expert with DNA1000 or DNA7500 Kit (Agilent). Sequences of the PCR primers used are available in Supplementary Table 2. Sequences of the PCR products were confirmed by direct sequencing or by cloning and sequencing. Statistical significance was assessed by one-way ANOVA followed by Dunnett’s post-hoc test versus RBM20^WT^ or Tukey-Kramer test by using R.

### Immunoprecipitation and phosphatase treatment

HEK293T cells expressing FLAG-tagged RBM20 proteins were lysed either with 5:1 mixture of RIPA buffer (Thermo) and 5 M NaCl (Nacalai) on ice or with SDS sample buffer at 95 °C. The lysates were sonicated in a water bath (UCD-300, Cosmo Bio), and the FLAG-RBM20 proteins were immuno-precipitated with anti-FLAG M2 Magnetic Beads (Sigma).

For calf intestine alkaline phosphatase (CiAP, Takara) treatment, the FLAG-RBM20 proteins on the magnetic beads were incubated with the enzyme in CiAP buffer at 37 °C for 90 min. For λ protein phosphatase (λPP, NEB) treatment, the beads were incubated in λPP buffer at 30 °C for 90 min. Heat-inactivation of the phosphatases were performed by pre-incubating the enzyme mixture at 65 °C for 1 hour. The FLAG-RBM20 proteins on the beads were then denatured with SDS or LDS sample buffer.

### Electrophoresis and staining of proteins

FLAG-tagged RBM20 proteins expressed in HEK293T cells or immuno-precipitated and subsequently treated with CiAP and λPP were separated by neutral polyacrylamide gel electrophoresis (NuPAGE, Invitrogen). The NuPAGE gel was stained with Pro-Q Diamond (Molecular Probes) according to the manufacturer’s instruction, and fluorescence images were acquired with FluoroPhore Star3000 (Anatech). The gel was then stained with Gel-Negative Staining Kit (Nacalai). For Phos-tag SDS-PAGE, a 15% polyacrylamide gel without or with 25 µM Phos-tag (Wako) and 50 µM MnCl_2_ was prepared and layered with a standard 4.5% stacking gel according to the manufacturer’s instruction. Protein samples were run with standard SDS running buffer (Nacalai). Vertical SDS-agarose gel electrophoresis of cardiac proteins from mice were performed essentially as described previously^37^. The proteins were detected by staining with CBB (Bio Craft). The images of the stained gels were captured with a scanner GT-X700 (Epson) and processed by using Photoshop CC (Adobe).

### Anti-phospho-RBM20 antibody

Rabbit polyclonal anti-phospho-RBM20 antiserum was raised with a synthetic phospho-peptide CYGPERPR(pS)R(pS)(Amd) by MBL, Nagoya, Japan. The serum was absorbed with a non-phospho-peptide CYGPERPRSRS(Amd) to yield TF1049–02 and further absorbed four times with a phospho-peptide CR(pS)R(pS)R(pS)R(pS) to yield TF1510-A.

### Western blot analysis

Proteins separated by standard SDS-PAGE, Phos-tag SDS-PAGE or NuPAGE were transferred to nitrocellulose membrane (Protran BA85, Whatman). The membranes were blocked with 5% skim milk and then incubated with 1 µg/ml anti-phospho-RBM20 (TF1049-02 or TF1510-A), anti-DDDDK-tag polyclonal antibody (MBL) or anti-FLAG monoclonal antibody (M2, Sigma) and 1:1,000-diluted HRP-conjugated anti-rabbit IgG antibody (Amersham or Pierce) or anti-mouse IgG antibody (MBL). Chemiluminescence signals (West Dura, Thermo) were detected by using LAS4000 (GE Healthcare).

### Knock-in mouse

*Rbm20*^*S637A*^ knock-in mice were generated by utilizing cloning-free CRISPR/Cas system as described previously^38^. The sequences of the crRNA and tracrRNA are 5′-CUCAUUGGACUUCGAGAACGGUUUUAGAGCUAUGCUGUUUUG-3′ and 5′-AAACAGCAUAGCAAGUUAAAAUAAGGCUAGUCCGUUAUCAACUUGAAAAAGUGGCACCGAGUCGGUGCU-3′, respectively, where an *Rbm20*-specific sequence is underlined. The sequence of the oligo DNA donor for *Rbm20*^*S637A*^ knock-in is 5′-TGCAGGTTACGAGCTCTGCAGAGTCTAAACCCTGTCTCTTCCCTTCCTCCCAGGTATGGTCCAGAGCGGCCACGTGCTCGAAGTCCAATGAGCCGATCACTCTCCCCAAGATCCCATAGTCCCCCAGGCCCCTCTCGGGCTGACTGGGGC-3′. The oligo RNAs and the DNA donor were chemically synthesized and purified by high pressure liquid chromatography (FASMAC, Japan). All care and experimental procedures of animals were in accordance with the guidelines for the Care and Use of Laboratory Animals published by National Research Council (The National Academy Presses, eighth edition, 2011) and subjected to a prior approval by the Institutional Animal Care and Use Committee of TMDU (Approval #A2017-080C).

### Data availability

The datasets generated during and/or analyzed during the current study are available from the corresponding authors on reasonable request.

## Results

### Identification of RBM20 mutations in DCM patients

We searched cohorts of 43 and 50 unrelated DCM patients for *RBM20* mutations and identified two (2.2%) mutations R634W and G1031X in Japan and USA, respectively (Table 1). The patient with heterozygous p.Arg634Trp (R634W) mutation: 18-years-old Japanese male patient with DCM. His affected father, and not unaffected mother, carried the same R634W mutation in a heterozygous state. The patient with homozygous p.Gly1031ter (G1031X) mutation: 21-years-old African American male patient with seizures, aborted sudden cardiac death (SCD) associated with prolonged QTc interval and severely reduced left ventricular (LV) function, compatible with the diagnosis of DCM, more specifically left ventricular non-compaction (LVNC). His parents did not show any cardiac symptoms, but his mother carried the G1031X mutation in a heterozygous state without any echocardiographic abnormalities (Table 1). A detailed genome analysis suggested a uniparental origin of the G1031X mutation in the patient. Detailed phenotypes of the patients will be reported elsewhere. Although these mutations were reported previously in other DCM patients^19,20^, their relevance to DCM remains unknown.

**Table 1:** Clinical characteristics of individuals carrying *RBM20* mutations.

ID	Mutation	Age, gender	Age at onset	Clinical diagnosis	Age at clinical exam	FH	NYHA	LVDd (mm)	LVDs (mm)	IVST (mm)	PWT (mm)	%FS	%EF	Other remarks
CM701	Arg634Trp	18 yo, male	18 yo	DCM	24 yo (34 yo)*	+	III	76 (88)*	69 (70)*	NA (10)*	NA (10)*	9 (20)*	16 (35)	Proband, SCD at the age of 34
CM702	Arg634Trp	43 yo, male	43 yo	DCM	43 yo	+	III	76	65	9	9	14	25–30	Father of CM701
MG15	Gly1031Ter	21 yo, male	21 yo	LVNC	21 yo	−	II	50	44	10	10	12	30	Proband, Ventricular hypokinesis, AF, QTc = 557 ms
MG13	Gly1031Ter	57 yo, female	N/A	Hyperlipidemia	57 yo	−	I	43	21	14	13	51	63.2	Mother of MG15

Abbreviations. yo, years-old; DCM, dilated cardiomyopathy; LVNC, left ventricular non-compaction; FH, family history of cardiomyopathy and/or sudden cardiac death; NYHA, classification of New York Heart Association; LVDd, diastolic left ventricular dimension; LVDs, systolic left ventricular dimension; IVST, intraventricular septum thickness; PWT, posterior wall thickness; %FS, % fractional shortening; %EF, % ejection fraction; SCD, sudden cardiac death; AF, atrial fibrillation; QTc, corrected QT interval in electrocardiogram; NA, not available.

*Data at the age of 34 are in parentheses.

The R634W mutation was registered as a single nucleotide polymorphism (ID no. rs796734066) in the dbSNP database^39^, but it was not found in a nucleotide sequence database from general populations, 1000 genomes^40^, exome aggregation consortium^41^ or human genetic variation database^42^. The R634W mutation was predicted to cause functional alteration by using *in silico* prediction programs: disease-causing by Mutation Taster, probably damaging (score 1.000) by PolyPhen-2, damaging (score 0) by SIFT, deleterious (score −3.8) by PROVEAN, and damaging (score −3.37) by FATHMM. Although the R634W mutation was noted to be conflicting in the ClinVar database^33^, the R634W and G1031X were classified as likely pathogenic and pathogenic, respectively, by the ACMG criteria^34^.

### Dichromatic fluorescence alternative splicing reporter for *Ttn* to monitor regulation activity of RBM20

**Figure 1:**
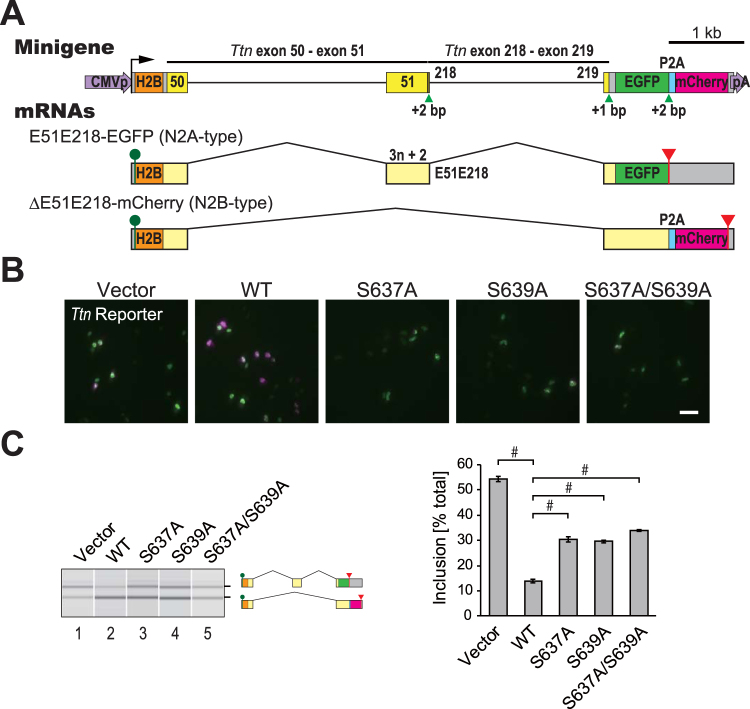
Dichromatic fluorescence alternative splicing reporter for Ttn to monitor the RBM20 activity in splicing regulation. (**A**) Schematic representation of the *Ttn* reporter minigene *TtnE50-E51E218-E219-EGFP/mCherry* (top) and mRNAs derived from it (bottom). Two genomic fragments *Ttn* E50-E51 and E218-E219 were inserted between human histone H2B cDNA and the EGFP/mCherry cassette. Expression of E51E218-EGFP and ΔE51E218-mCherry indicates inclusion and skipping of a chimeric exon E51E218, respectively. (**B**) Microphotographs of HeLa cells co-transfected with the fluorescence *Ttn* splicing reporter minigene and an empty vector or an expression vector for the wild-type (WT) or mutant RBM20 protein. Fluorescence of EGFP and mCherry is pseudo-colored in green and magenta, respectively. Scale bar, 100 µm. (**C**) RT-PCR analysis of the *Ttn* splicing reporter minigene co-expressed with an empty vector or an expression vector for the wild-type (WT) or mutant RBM20 protein in HeLa cells. Representative gel-like presentation (left) and calculated inclusion levels (right) are indicated. Error bars indicate standard errors of the means. ^#^p < 0.001 and **p < 0.01 to WT (n = 3 biological replicates, one-way ANOVA followed by Dunnett’s test).

In order to evaluate the activity of RBM20 as a critical pre-mRNA splicing regulator for producing titin N2B isoform, we intended to construct a dichromatic *Ttn* alternative splicing (AS) reporter minigene. Among many AS events in the *Ttn* pre-mRNA processing, we focused on two critical introns, intron 50 and intron 218, both of which must not be excised during production of the titin N2B mRNA. The minigene *TtnE50-E51E218-E219-EGFP/mCherry* (Fig. 1A) carries two genomic fragments spanning from exon 50 (E50) through exon 51 (E51) and from exon 218 (E218) through exon 219 (E219), and the fragments are connected so that E51 and E218 are fused to form a 590-nt chimeric exon (E51E218). The *Ttn* fragments were cloned upstream of an EGFP/mCherry cassette where a frameshift was introduced between green (EGFP) and red (mCherry) fluorescent protein cDNAs^43,44^. The two fluorescent proteins are produced in a mutually exclusive manner upon alternative splicing of the chimeric exon E51E218 (Fig. 1A); EGFP is expressed when the chimeric exon is included, while mCherry is expressed by N2B-type splicing where E50 is directly connected to E219.

To examine whether the *TtnE50-E51E218-E219-EGFP/mCherry* reporter recapitulates RBM20-dependent N2B-type spicing, we co-transfected the reporter minigene with an empty vector or an expression vector for a full-length mouse RBM20, and analyzed splicing patterns of minigene-derived mRNAs by semi-quantitative reverse transcription (RT)-polymerase chain reaction (PCR). Fluorescence of both EGFP and mCherry was detected in cells co-transfected with the empty vector (Fig. 1B, vector) and expression of the two expected mRNAs were confirmed (Fig. 1C, lane 1). Co-expression of wild-type RBM20 promoted expression of ΔE51E218-mCherry and repressed expression of E51E218-EGFP (Fig. 1B, WT and Fig. 1C, lane 2), consistent with its activity to promote N2B-type splicing. When conserved serine residues in the RSRSP stretch (Ser637 and/or Ser639, corresponding to Ser635 and Ser637, respectively, in human RBM20) were replaced with alanine, the splicing regulation activity was significantly affected (Fig. 1B,C, lanes 3–5). These results indicated that the *TtnE50-E51E218-E219-EGFP/mCherry* reporter could recapitulate RBM20-mediated regulation of the *Ttn* pre-mRNA processing and could be utilized to validate functional alterations caused by mutations in the RBM20 protein.

### The RRM domain and the zinc finger domains of RBM20 are dispensable for splicing regulation of the reporter

**Figure 2:**
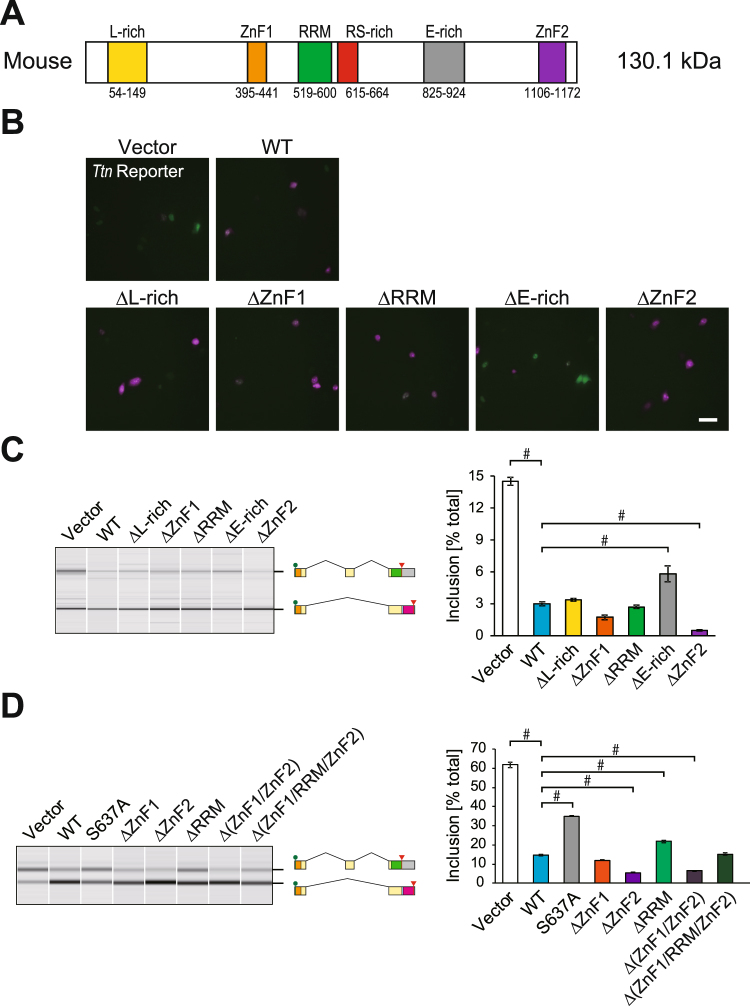
The RRM domain and the zinc finger domains of RBM20 are not critical for splicing regulation of the *Ttn* reporter. (**A**) Domain structure of mouse RBM20 protein. Names and positions of the domains are indicated. These domains are deleted in the mutants used in panels (**B–D**). (**B**) Microphotographs of HeLa cells co-transfected with the fluorescence *Ttn* splicing reporter minigene and an expression vector for the wild-type (WT) or deletion mutant RBM20 protein. The images are presented as in Fig. 1B. (**C**,**D**) RT-PCR analysis of the *Ttn* splicing reporter minigene co-expressed with an expression vector for the wild-type (WT) or mutant RBM20 protein in HeLa cells. The data are presented as in Fig. 1C.

Because RBM20 protein consists of multiple domains (Fig. 2A), we next asked which domain(s) is/are critical for the N2B-type splicing of *Ttn*. To this end, we constructed expression vectors for RBM20 mutants in which each domain was deleted. Co-expression with the fluorescence *Ttn* splicing reporter revealed that a glutamate (E)-rich region was required for the efficient splicing regulation, whereas putative RNA-binding domains, an RRM domain and two ZnF domains, were dispensable for the splicing regulation (Fig. 2B). Semi-quantitative RT-PCR analysis confirmed the observation (Fig. 2C). To further assess requirement of these domains, we constructed ZnF1/ZnF2 double and ZnF1/RRM/ZnF2 triple deletion mutants and found that these mutants repressed the *Ttn* reporter exon as effectively as the wild type or the single mutants (Fig. 2D). These data indicated that the putative RNA-binding domains were dispensable at least for the N2B-type splicing of the *Ttn* splicing reporter minigene and highlighted an essential role for the RSRSP stretch.

### The RSRSP stretch is critical for nuclear localization of and not for splicing regulation by RBM20

**Figure 3:**
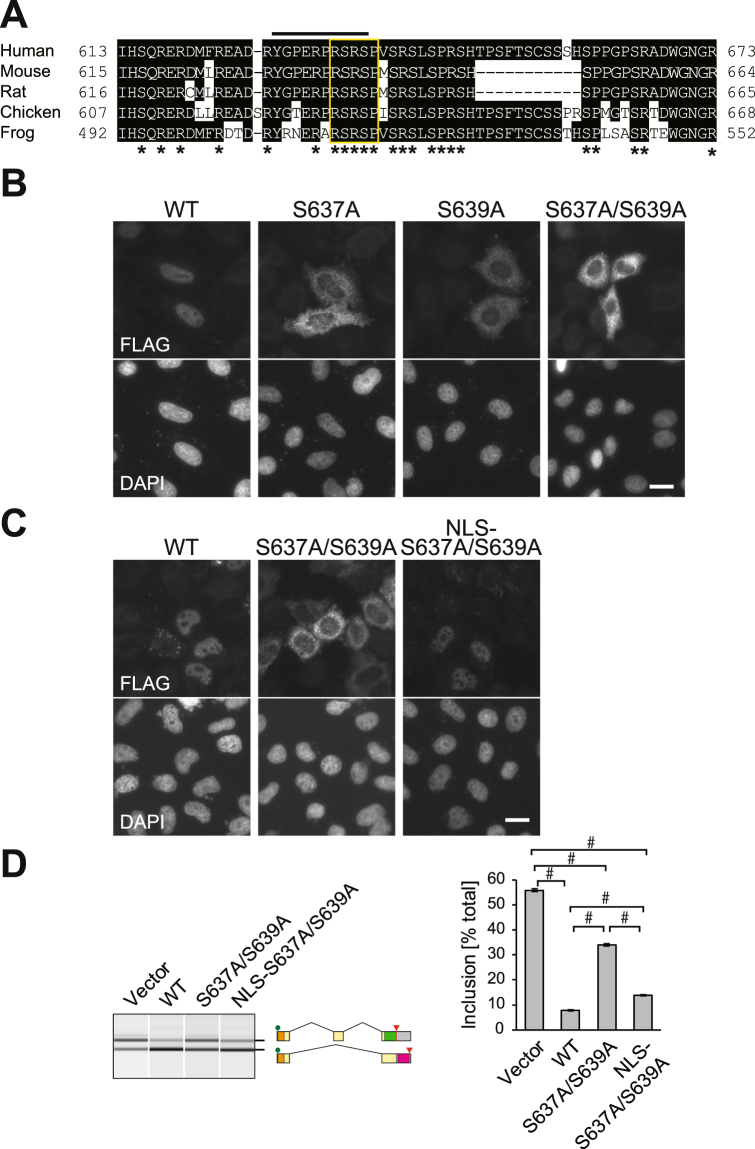
The RSRSP stretch is critical for nuclear localization of and not splicing regulation by RBM20. (**A**) Amino acid sequence alignment of the RS-rich region of RBM20 proteins from human (Accession No. NP_001127835), mouse (NP_001164318), rat (NP_001101081), chicken (XP_015144459) and frog (XP_002942947) by Clustal W and manual adjustment. Amino acid residues that match the human RBM20 residues are shaded. The RSRSP stretch is boxed. Asterisks indicate evolutionarily conserved arginine (R), serine (S) and proline (P) residues. A line above the alignment indicates the sequence of a phospho-peptide used for raising anti-phospho-RBM20 antibody. (**B**) Immunofluorescence staining of FLAG-tagged wild-type RBM20 and substitution mutants RBM20^S637A^, RBM20^S639A^ and RBM20^S637A/S639A^ ectopically expressed in HeLa cells. DAPI staining of the same field is indicated below each panel. Scale bar, 20 µm. (**C)** Immunofluorescence staining of FLAG-tagged wild-type RBM20, RBM20^S637A/S639A^ and RBM20^S637A/S639A^ with three tandem copies of SV40 NLSs (NLS-RBM20^S637A/S639A^) in HeLa cells. The images are shown as in (**B**). (**D**) RT-PCR analysis of the *Ttn* splicing reporter minigene co-expressed with an expression vector for wild-type (WT) RBM20, RBM20^S637A/S639A^ and NLS-RBM20^S637A/S639A^ in HEK293T cells. The data are presented as in Fig. 1C. Significance of differences in the mean inclusion levels was analyzed as indicated by one-way ANOVA followed by Tukey-Kramer test.

In order to reveal the role for the RSRSP stretch within the RS-rich region (Figs 2A and 3A) in the splicing regulation by RBM20, we analyzed effects of mutations in the stretch on subcellular localization of RBM20, because arginine/serine-rich (RS) domains of SR proteins, a well-characterized family of splicing factors, are known to play roles in their nuclear localization^45,46^. Wild-type (WT) RBM20 ectopically expressed in HeLa cells were localized in nuclei in most cells (Fig. 3B), consistent with its function as a pre-mRNA splicing regulator. Strikingly, the substitution mutants RBM20^S637A^, RBM20^S639A^ and RBM20^S637A/S639A^ were excluded from the nuclei (Fig. 3B), indicating that these residues are essential for the nuclear localization.

To ask whether the RSRSP stretch is required only for the nuclear localization or is involved in the splicing regulation as well, we forced to localized the RBM20^S637A/S639A^ mutant protein by adding nuclear localization signals (NLSs). We confirmed nuclear localization of NLS-RBM20^S637A/S639A^ (Fig. 3C) and the splicing regulation of the *Ttn* reporter minigene was restored (Fig. 3D). These results indicated that the RSRSP stretch was critical for nuclear localization of and not splicing regulation by RBM20.

### RBM20 is constitutively phosphorylated on the RSRSP stretch and some other residue(s)

**Figure 4:**
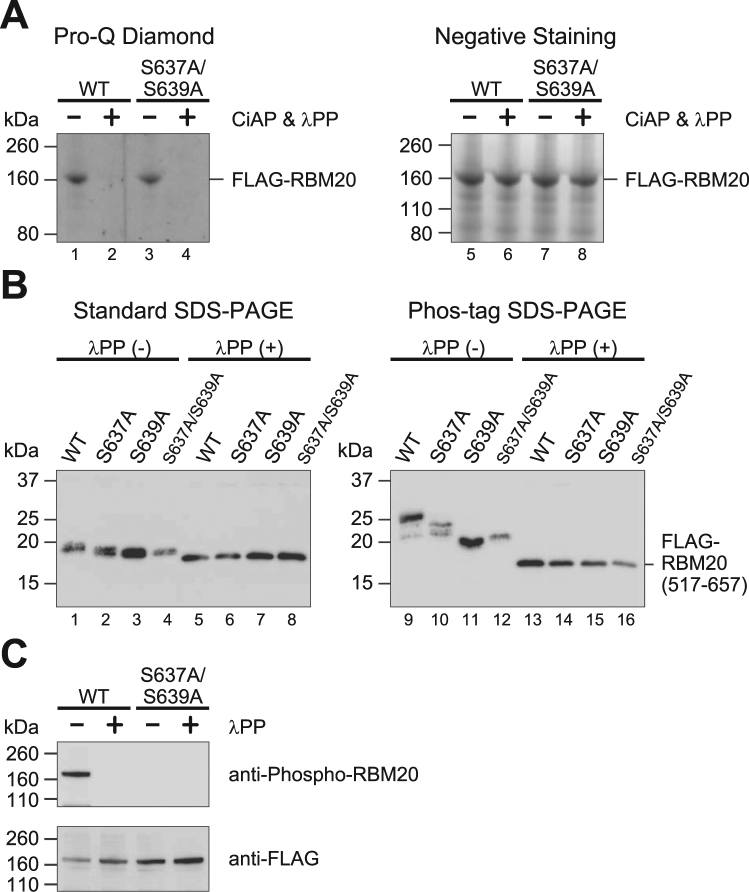
RBM20 is constitutively phosphorylated on the RSRSP stretch and some other site(s). (**A**) Pro-Q Diamond staining (left) and negative staining (right) of immuno-precipitated and polyacrylamide gel-electrophoresed FLAG-tagged RBM20^WT^ and RBM20^S637A/S639A^ expressed in HEK293T cells. The precipitated proteins were sequentially incubated with heat-inactivated (−) or active (+) calf intestine protein phosphatase (CiAP) and lambda protein phosphatase (λPP). (**B**) Western blot detection of immuno-precipitated FLAG-tagged wild-type RBM20(517–657), RBM20(517–657)^S637A^, RBM20(517–657)^S639A^ and RBM20(517–657)^S637A/S639A^ expressed in HEK293T cells. The proteins were separated by either standard SDS-PAGE (left) or SDS-PAGE with 25 µM Phos-tag (right) following incubation without (−) or with (+) λPP. (**C**) Western blot detection of immuno-precipitated FLAG-tagged full-length RBM20 and RBM20^S637A/S639A^ expressed in HEK293T cells following incubation without (−) or with (+) λPP. Antibodies used are indicated on the right.

Given the similar functional properties of the arginine/serine-rich regions in RBM20 and the SR proteins, we hypothesized that the RSRSP stretch of RBM20 was phosphorylated for nuclear localization like the RS domains of the SR proteins^45,47,48^. We therefore purified RBM20^WT^ and RBM20^S637A/S639A^ ectopically expressed in HEK293T cells and stained phosphoproteins with Pro-Q Diamond after polyacrylamide gel electrophoresis. Both RBM20^WT^ and RBM20^S637A/S639A^ were stained to apparently the same extent (Fig. 4A, lanes 1 and 3), and the signals were completely lost by pre-incubation with active phosphatases (Fig. 4A, lanes 2 and 4). These results indicated that RBM20 was indeed a phosphoprotein and was phosphorylated on residue(s) outside the RSRSP stretch.

We then focused on the RS-rich region to identify the phosphorylated residues by utilizing expression vectors for RBM20(517–657) possessing only the RRM domain and the RS-rich region. Wild-type and mutant versions of the truncated RBM20 protein were immuno-precipitated, incubated without or with lambda phosphatase (λPP), and separated by standard or phosphate-affinity SDS-PAGE^49^. Under a standard SDS-PAGE condition, all the truncated proteins migrated to a similar extent, although the untreated proteins were slightly retarded (Fig. 4B, lanes 1–8). In the presence of 25 µM Phos-tag, mobility of the wild-type RBM20(517–657) was largely retarded (lane 9), indicating that RBM20(517–657)^WT^ was constitutively phosphorylated. Substitution mutations at positions of Ser637 and/or Ser639 decreased the mobility shift by Phos-tag (lanes 10–12), indicating that these mutations diminished phosphorylation levels. λPP treatment of the wild-type and the mutant RBM20(517–657) proteins further increased the mobility of all these proteins to the same level equivalent to those in the standard conditions (lanes 5–8 and 13–16), indicating that the mutant proteins were still phosphorylated on other residue(s).

To confirm phosphorylation of the Ser637 and Ser639 residues in the RSRSP stretch, we raised phosphorylation-specific polyclonal antibodies against a peptide sequence fully conserved in mammals (Fig. 3A). The anti-phospho-RBM20 antibody detected full-length wild-type RBM20 and not RBM20^S637AS639A^ in a λPP-sensitive manner (Fig. 4C). Taken together, our results demonstrate that RBM20 was highly and constitutively phosphorylated on multiple residues including the two serine residues in the RSRSP stretch.

### Rbm20^S637A^ knock-in mice are defective in generating the N2B isoform of titin in the heart

**Figure 5:**
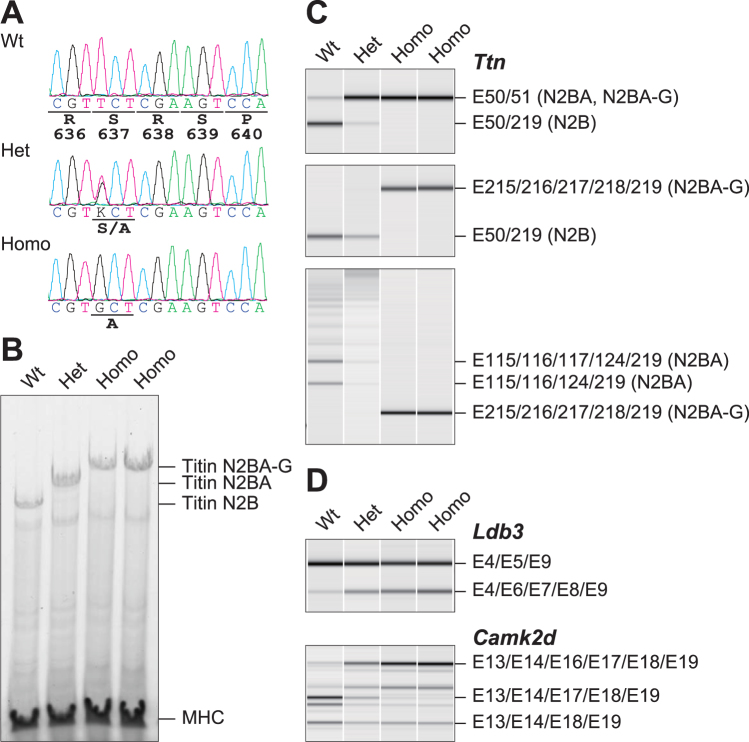
*Rbm20*^*S637A*^ knock-in mice are defective in producing the N2B isoform of titin in the heart. (**A**) Sequence analysis of *Rbm20* mRNAs from hearts of a wild-type mouse (Wt) and a heterozygote (Het) and a homozygote (Homo) of *Rbm20*^*S637A*^ knock-ins. (**B**) Vertical SDS-agarose gel electrophoresis and CBB staining of cardiac proteins from the hearts of 7-week-old *Rbm20*^*S637A*^ knock-in mice in one litter. Genotypes of the individual mice are indicated above. Titin isoforms (N2B, N2BA and N2BA-G) and myosin heavy chain (MHC) are indicated. (**C**) RT-PCR analysis of *Ttn* mRNAs in the mice shown in (**B**) with E50 forward, E51 reverse and E219 reverse primers (top), E50 forward, E215 forward and E219 reverse primers (middle) and E115 forward, E215 forward and E219 reverse primers (bottom). Splicing patterns of the PCR products and names of corresponding titin isoforms are indicated on the right. (**D**) RT-PCR analysis of *Ldb3* (top) and *Camk2d* (bottom) mRNAs as shown in (**C**).

To confirm functional reduction of RBM20 by mutations in the RSRSP stretch, we generated *Rbm20*^*S637A*^ knock-in mice (Fig. 5A) by using a cloning-free CRISPR/Cas system^38^. We first analyzed titin isoform expression in the hearts from heterozygous and homozygous *Rbm20*^*S637A*^ mice as well as a littermate control mouse. As expected, the cardiac titin proteins from the heterozygote migrated more slowly than those from the wild type; those from the homozygotes migrated further slowly (Fig. 5B). We then analyzed the splicing patterns of the endogenous *Ttn* mRNAs (Fig. 5C). The N2B isoform (as demonstrated by E50/E219 splicing) predominated over the N2BA isoforms (E50/E51 splicing) in the wild type, whereas the N2BA-type splicing predominated in the heterozygote and the N2B isoform was undetectable in the homozygotes (Fig. 5C, top panel). The isoform expressed in the homozygote was considered to be giant N2BA (N2BA-G)^6,50^ because it included exons 215–218 that were skipped in the wild type and even in the heterozygote (Fig. 5C, middle panel). Among the N2BA isoforms, two short isoforms (E115/E116/E117/E124/E219 and E115/E116/E124/E219 splicing) were predominant in the wild type and not in the heterozygote (Fig. 5C, bottom panel), suggesting that longer N2BA isoforms were produced in the heterozygote. These results were consistent with a report on a rat model of *Rbm20* gene deletion^6^ and a mouse model of *Rbm20*^*ΔRRM*^ lacking the RRM domain^51^. Alternative splicing of two other known target genes *Ldb3* and *Camk2d*^6,51^ were also affected in the knock-in mice (Fig. 5D), indicating that the single amino acid substitution in the RSRSP stretch eliminated the function of RBM20 and dramatically affected isoform expression of the cardiac titin even in the heterozygous state.

### Evaluation of functional alterations caused by RBM20 mutations identified in DCM patients

**Figure 6:**
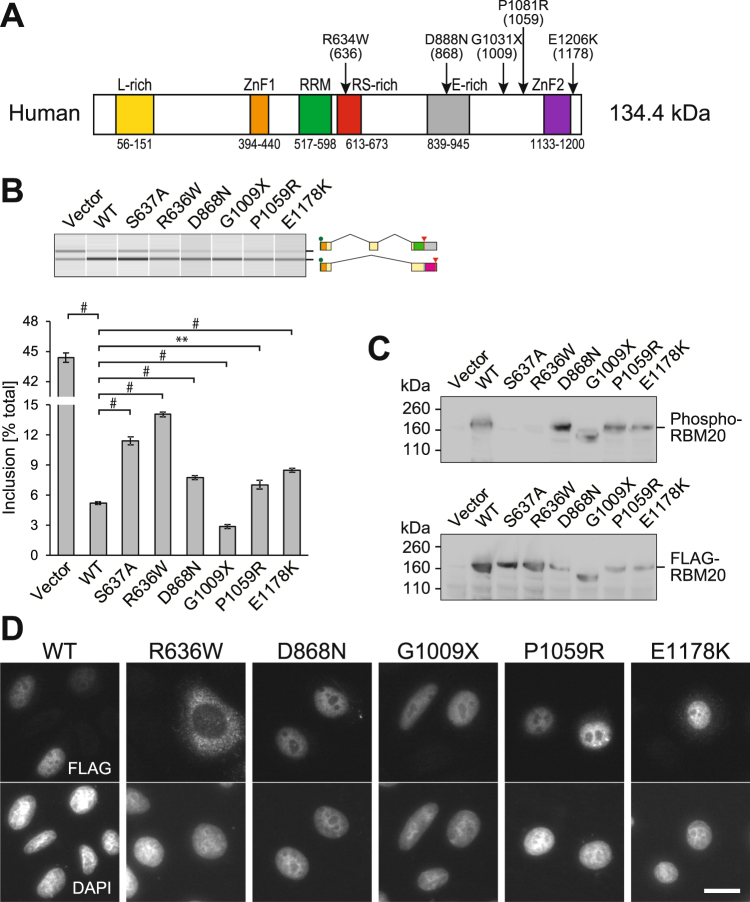
Evaluation of *RBM20* mutations identified in DCM patients. (**A**) Point mutations in the coding region of the human *RBM20* gene analyzed in this study. Domain structure of the human RBM20 protein and positions of the mutations are indicated. Numbers in parentheses indicate positions of corresponding residues in the mouse RBM20 protein. (**B**) RT-PCR analysis of the *Ttn* splicing reporter minigene co-expressed with an expression vector for the wild-type (WT) or mutant RBM20 protein in HeLa cells. The data are presented as in Fig. 1C. (**C**) Western blot analysis of FLAG-tagged wild-type and mutant RBM20 proteins ectopically expressed in HEK293T cells with anti-phospho-RBM20 (top) and anti-FLAG (bottom) antibodies. (**D**) Immunofluorescence staining of FLAG-tagged wild-type and mutant RBM20 proteins ectopically expressed in HeLa cells. DAPI staining of the same field is indicated below each panel. Scale bar, 20 µm.

With the tools we developed in this study, we evaluated the functional relevance of mutations in the coding region of *RBM20* we found and/or reported in DCM patients^19^. The *RBM20* mutations analyzed in this study are summarized in Fig. 6A. We introduced point mutations at the corresponding positions in the mouse RBM20 expression vector and analyzed the effects of the mutations on splicing regulation of the *Ttn* splicing reporter (Fig. 6B), phosphorylation status of the RSRSP stretch (Fig. 6C), and subcellular localization of RBM20 (Fig. 6D).

Not surprisingly, R636W substitution (equivalent to R634W in human), affecting the first arginine residue in the RSRSP stretch, diminished splicing regulation activity of RBM20 as did the S637A substitution (Fig. 6B). The RBM20^R636W^ protein was almost undetectable with the anti-phospho-RBM20 antibody (Fig. 6C) and was excluded from the nuclei (Fig. 6D), consistent with the idea that phosphorylation of the RSRSP stretch is necessary for nuclear localization of RBM20. The other tested substitutions, D868N, P1059R and E1178K, which were equivalent to D888N, P1081R and E1206K in human mutations, respectively, had less effect on RBM20 properties at least under our experimental conditions (Fig. 6B,D). A truncation mutation G1009X (equivalent to G1031X in human) changed the splicing of the *Ttn* reporter slightly more effectively than wild-type RBM20 (Fig. 6B,D) like a deletion mutant lacking the second ZnF domain (Fig. 2C,D).

## Discussion

In the present study, we screened panels of DCM patients and identified two known *RBM20* mutations, R634W and G1031X, in Japanese and African American patients, respectively. To assess the functional relevance of *RBM20* mutations and biological role of functional domains of RBM20, we developed a fluorescence *Ttn* splicing reporter assay, and demonstrated that the two serine residues in the RSRSP stretch of RBM20 were constitutively phosphorylated and served as a critical part of NLS. Hence, mutations in any residue in the RSRSP stretch, a hotspot of DCM mutations, lead to mislocalization of RBM20 and may result in dysregulation of alternative splicing of the *TTN* gene and other target genes. On the other hand, G1031X showed an enhanced splicing activity in the *Ttn* mini-gene system.

As demonstrated here and in the literature, RBM20 is a crucial splicing regulator in the adult heart; even heterozygous mutations greatly affect titin isoform expression and cause clinical symptoms^5,6,51^. Paradoxically, the embryonic heart and skeletal muscles express long isoforms of titin, fetal N2BA and N2A, respectively, which include many or all of exons 51–218^52–54^ even though RBM20 is expressed^55,56^. The ratio of the titin isoforms varies not only during development^14,52,53,56,57^, but also among different muscles^54^, from species to species^58,59^, due to artificial volume overload to the left ventricle^16^ and genetic manipulation of *Ttn*^60^; all of these are dependent on RBM20. Such flexible regulation of RBM20-mediated alternative splicing were explained by (i) other factors co-acting with and/or antagonizing RBM20^55,61^ and (ii) signal transduction or conditions leading to changes in the amount of RBM20 at a transcript^18^ or protein^62,63^ level. Our present study provides insights into another layer of RBM20 regulation; i.e. reversible subcellular localization of RBM20 by a phosphorylation-dephosphorylation cycle. It is therefore intriguing to analyze phosphorylation status of endogenous RBM20 in developing heart as well as to monitor shuttling into and out of the nucleus in a future study.

The R634W mutation we and others found in DCM patients (R636W in mouse), affecting the first arginine residue in the RSRSP stretch, diminished the function of RBM20 to an extent similar to that of the S635A mutation (S637A in mouse) in our splicing reporter assay (Fig. 6). We found that these mutant RBM20 proteins were excluded from the nucleus (Figs 3 and 6), consistent with a previous quantitative proteomic analysis showing that S635A mutation in human RBM20 significantly reduced interaction with 38 splicing-related proteins^18^. Fatal effects of these mutations as well as other DCM-related mutations in the hotspot of *RBM20* is likely due to loss of recognition of the RSRSP stretch by protein kinase(s) that phosphorylate the serine residues or by a partner protein that specifically binds to RBM20 only after phosphorylation of the RSRSP stretch. Loss of the second ZnF domain by the G1031X mutation we and others identified in DCM patients (G1009X in mouse) (Fig. 6) or by targeted deletion (Fig. 2) unexpectedly enhanced the repressor activity of RBM20 at least in our reporter assay. This may be due to destabilization or sequestration activity of the second ZnF domain and may depend on specific conditions of the assay such as reporter structure and/or cell types used. Either way, loss of the second ZnF domain affected certain aspects of the RBM20 functions. The DCM patient we reported here was apparently homozygous for the G1031X mutation, while his unaffected mother was heterozygous, indicating that the effect of this mutation, unlike the other familial mutations, is recessive if any. It is interesting to examine whether the enhanced splicing would be applicable to other target genes reported to be regulated by RBM20 in the heart^6,18^, but we could not obtain heart biopsy samples from the carrier of G1031X, because biopsy was denied by them. Therefore, this issue should be investigated by using, for example, cardiac cell lines homozygously knocked-in the G1031X mutation in the future study.

The other DCM-related missense mutations we analyzed in this study, D888N, P1081R and E1206K (D868N, P1059R and E1178K in mouse *Rbm20*, respectively)^19^, only moderately affected the function of RBM20 (Fig. 6). The differences in the effects of these potentially pathogenic mutations on RBM20 functions might explain differences in clinical symptoms among the DCM patients although further family study or animal models are necessary to obtain genetic evidence. It should be noted here that D888N variant was found in ExAc database at 0.284%, registered in dbSNP database as rs796734066, listed as ‘conflicting’ in ClinVar database. Its *in silico* prediction data were disease-causing (Mutation Taster), probably damaging (PolyPhen-2 score 0.954), damaging (SIFT score 0.001), neutral (PROVEAN score −1.92) and damaging (fathmm score −2.84). On the other hand, P1081R and E1206K variants were found at less than 0.01%, not registered in dbSNP or ClinVar database, while their *in silico* prediction data showed both were polymorphisms (Mutation Taster) and neutral (PROVEAN). In addition, P1081R was predicted to be benign (PolyPhen-1 score 0.183) and tolerated (fathmm score −0.86), whereas E1206K was predicted to be possibly damaging (PolyPhen-2 score 0.829), damaging (SIFT score 0.001) and damaging (fathmm score −3.12). Therefore, the pathological significance of these variants remain to be further clarified.

A recent study reported a family with DCM that has a heterozygous missense mutation in the E-rich region (*RBM20*^*E913K/*+^)^64^. This is consistent with our finding that the E-rich region was the only domain whose deletion diminished the RBM20 functions (Fig. 2). An in-frame deletion of the RRM domain in mouse models resulted in similarly severe splicing defects as in the *Rbm20* deletion rat model^51^, whereas deletion of the RRM domain had minimal effect in our reporter assay (Fig. 2). This may be explained by two distinct functions for RBM20 implicated by careful analysis of RT-PCR products in the *Rbm20* deletion rat strain and tethering of RBM20 in reporter assays^55^: (i) to locally bind to RBM20-repressed regions and ii) to mediate aggregation of such RBM20 proteins to fully repress clusters of exons. Indeed, crosslinking and immunoprecipitation coupled with high-throughput RNA sequencing (CLIP-seq) analysis revealed that RBM20 directly bound to UCUU-containing elements in flanking introns of its target exons in living cells^18^, yet it was not demonstrated which domains were directly involved in the specific recognition of the elements. The RRM domain may play critical roles in aggregating multiple RBM20 proteins to effectively repress multiple exons as in the *Ttn*, *Ldb3* and *Camk2d* pre-mRNAs and not in repressing a single exon as in our reporter assay.

This study has demonstrated for the first time that RBM20 is phosphorylated in cells and that its phosphorylation on the RS-rich region is critical for its nuclear localization. A previous study on subcellular localization of RBM20 revealed that the RRM domain and the RS-rich region were required for nuclear retention^65^, yet did not identify a critical residue. We demonstrated that either of the two serine residues in the RSRSP stretch was critical. Our data in Fig. 4B suggests order of phosphorylation; Ser637 phosphorylation depends on Ser639 as the mobility shift of RBM20(517–657)^S639A^ in the Phos-tag gel was equivalent to that of RBM20(517–657)^S637A/S639A^, whereas Ser639 could be phosphorylated in the S637A mutant as the mobility shift of RBM20(517–657)^S637A^ was in between those of RBM20(517–657)^WT^ and RBM20(517–657)^S637A/S639A^. Other residues outside of the RSRSP stretch were phosphorylated even in the absence of Ser637 and Ser639 (Fig. 4A,B), emphasizing that simultaneous phosphorylation of both of the serine residues is critical for the nuclear localization.

The amino acid composition and function of the RS-rich region of RBM20 are similar to those of the RS domains of the SR-family splicing factors^46,66–68^ in that they i) are rich in RS, SR and serine-proline (SP) dipeptides (Fig. 3A), (ii) reside C-terminally to RRM domain(s) (Figs 2A and 6A), (iii) are extensively phosphorylated in cells (Fig. 4), and iv) serve as NLSs (Fig. 3). Substantial differences, however, are also evident: the SR proteins bind to exonic elements and promote splicing in general, whereas RBM20 binds to intronic elements and mainly represses splicing^18^; the number of the RS dipeptides in the only consecutive RS repeats, constituting the RSRSP stretch, is only two in RBM20 (Fig. 3A), whereas the SR proteins have multiple longer RS repeats. Phosphorylation of the RS domains facilitate protein-protein interaction and is required for splicing^45,69^, whereas the RSRSP stretch of RBM20 appears to be required only for nuclear localization (Fig. 3). Our study implies that phosphorylation and likely dephosphorylation of the RSRSP stretch is the site of reversible regulation of RBM20 activity in the heart. It is therefore interesting to identify a protein kinase and an importin protein for RBM20 *in vivo*, which will elucidate physiological and pathological relevance of the phosphorylation of RBM20.

## Electronic supplementary material

Supplementary Information

## References

[1] Hershberger RE, Siegfried JD (2011). Update 2011: clinical and genetic issues in familial dilated cardiomyopathy. Journal of the American College of Cardiology.

[2] McCartan C, Mason R, Jayasinghe SR, Griffiths LR (2012). Cardiomyopathy classification: ongoing debate in the genomics era. Biochem Res Int.

[3] Elliott P (2008). Classification of the cardiomyopathies: a position statement from the European Society Of Cardiology Working Group on Myocardial and Pericardial Diseases. European Heart Journal.

[4] Perez-Serra A (2016). Genetic basis of dilated cardiomyopathy. International Journal of Cardiology.

[5] Brauch KM (2009). Mutations in ribonucleic acid binding protein gene cause familial dilated cardiomyopathy. Journal of the American College of Cardiology.

[6] Guo W (2012). RBM20, a gene for hereditary cardiomyopathy, regulates titin splicing. Nature Medicine.

[7] Labeit S, Kolmerer B (1995). Titins: giant proteins in charge of muscle ultrastructure and elasticity. Science.

[8] Gigli M (2016). A Review of the Giant Protein Titin in Clinical Molecular Diagnostics of Cardiomyopathies. Front Cardiovasc Med.

[9] Bang ML (2001). The complete gene sequence of titin, expression of an unusual approximately 700-kDa titin isoform, and its interaction with obscurin identify a novel Z-line to I-band linking system. Circulation Research.

[10] Cazorla O (2000). Differential expression of cardiac titin isoforms and modulation of cellular stiffness. Circulation Research.

[11] Granzier HL, Irving TC (1995). Passive tension in cardiac muscle: contribution of collagen, titin, microtubules, and intermediate filaments. Biophysical Journal.

[12] Anderson BR, Granzier HL (2012). Titin-based tension in the cardiac sarcomere: molecular origin and physiological adaptations. Progress in Biophysics and Molecular Biology.

[13] LeWinter MM, Wu Y, Labeit S, Granzier H (2007). Cardiac titin: structure, functions and role in disease. Clinica Chimica Acta.

[14] Opitz CA, Leake MC, Makarenko I, Benes V, Linke WA (2004). Developmentally regulated switching of titin size alters myofibrillar stiffness in the perinatal heart. Circulation Research.

[15] LeWinter MM, Granzier HL (2014). Cardiac titin and heart disease. Journal of Cardiovascular Pharmacology.

[16] Hutchinson KR, Saripalli C, Chung CS, Granzier H (2015). Increased myocardial stiffness due to cardiac titin isoform switching in a mouse model of volume overload limits eccentric remodeling. Journal of Molecular and Cellular Cardiology.

[17] Makarenko I (2004). Passive stiffness changes caused by upregulation of compliant titin isoforms in human dilated cardiomyopathy hearts. Circulation Research.

[18] Maatz H (2014). RNA-binding protein RBM20 represses splicing to orchestrate cardiac pre-mRNA processing. Journal of Clinical Investigation.

[19] Refaat MM (2012). Genetic variation in the alternative splicing regulator RBM20 is associated with dilated cardiomyopathy. Heart Rhythm.

[20] Li D (2010). Identification of novel mutations in RBM20 in patients with dilated cardiomyopathy. Clinical and Translational Science.

[21] Millat G (2011). Clinical and mutational spectrum in a cohort of 105 unrelated patients with dilated cardiomyopathy. European Journal of Medical Genetics.

[22] Rampersaud E (2011). Rare variant mutations identified in pediatric patients with dilated cardiomyopathy. Progress in Pediatric Cardiology.

[23] Waldmuller S (2015). Targeted 46-gene and clinical exome sequencing for mutations causing cardiomyopathies. Molecular and Cellular Probes.

[24] Zhao Y (2015). Targeted next-generation sequencing of candidate genes reveals novel mutations in patients with dilated cardiomyopathy. International Journal of Molecular Medicine.

[25] Kuroyanagi H, Watanabe Y, Hagiwara M (2013). CELF family RNA-binding protein UNC-75 regulates two sets of mutually exclusive exons of the *unc-32* gene in neuron-specific manners in *Caenorhabditis elegans*. PLoS Genet.

[26] Kuroyanagi H, Ohno G, Mitani S, Hagiwara M (2007). The Fox-1 family and SUP-12 coordinately regulate tissue-specific alternative splicing *in vivo*. Molecular and Cellular Biology.

[27] Kuroyanagi H, Kobayashi T, Mitani S, Hagiwara M (2006). Transgenic alternative-splicing reporters reveal tissue-specific expression profiles and regulation mechanisms *in vivo*. Nat Methods.

[28] Adzhubei IA (2010). A method and server for predicting damaging missense mutations. Nat Methods.

[29] Kumar P, Henikoff S, Ng PC (2009). Predicting the effects of coding non-synonymous variants on protein function using the SIFT algorithm. Nature Protocols.

[30] Choi Y, Sims GE, Murphy S, Miller JR, Chan AP (2012). Predicting the functional effect of amino acid substitutions and indels. PloS One.

[31] Shihab HA (2013). Predicting the functional, molecular, and phenotypic consequences of amino acid substitutions using hidden Markov models. Human Mutation.

[32] Schwarz JM, Cooper DN, Schuelke M, Seelow D (2014). MutationTaster2: mutation prediction for the deep-sequencing age. Nat Methods.

[33] Landrum MJ (2014). ClinVar: public archive of relationships among sequence variation and human phenotype. Nucleic Acids Research.

[34] Richards S (2015). Standards and guidelines for the interpretation of sequence variants: a joint consensus recommendation of the American College of Medical Genetics and Genomics and the Association for Molecular Pathology. Genetics in Medicine.

[35] Kuroyanagi H, Watanabe Y, Suzuki Y, Hagiwara M (2013). Position-dependent and neuron-specific splicing regulation by the CELF family RNA-binding protein UNC-75 in *Caenorhabditis elegans*. Nucleic Acids Research.

[36] Kuroyanagi H, Ohno G, Sakane H, Maruoka H, Hagiwara M (2010). Visualization and genetic analysis of alternative splicing regulation *in vivo* using fluorescence reporters in transgenic *Caenorhabditis elegans*. Nature Protocols.

[37] Warren CM, Krzesinski PR, Greaser ML (2003). Vertical agarose gel electrophoresis and electroblotting of high-molecular-weight proteins. Electrophoresis.

[38] Aida T (2015). Cloning-free CRISPR/Cas system facilitates functional cassette knock-in in mice. Genome Biology.

[39] Sherry ST (2001). dbSNP: the NCBI database of genetic variation. Nucleic Acids Research.

[40] Genomes Project C (2015). A global reference for human genetic variation. Nature.

[41] Lek M (2016). Analysis of protein-coding genetic variation in 60,706 humans. Nature.

[42] Higasa K (2016). Human genetic variation database, a reference database of genetic variations in the Japanese population. Journal of Human Genetics.

[43] Orengo JP, Bundman D, Cooper TA (2006). A bichromatic fluorescent reporter for cell-based screens of alternative splicing. Nucleic Acids Research.

[44] Takeuchi A, Hosokawa M, Nojima T, Hagiwara M (2010). Splicing reporter mice revealed the evolutionally conserved switching mechanism of tissue-specific alternative exon selection. PloS One.

[45] Yeakley JM (1999). Phosphorylation regulates *in vivo* interaction and molecular targeting of serine/arginine-rich pre-mRNA splicing factors. Journal of Cell Biology.

[46] Caceres JF, Misteli T, Screaton GR, Spector DL, Krainer AR (1997). Role of the modular domains of SR proteins in subnuclear localization and alternative splicing specificity. Journal of Cell Biology.

[47] Wang HY (1998). SRPK2: a differentially expressed SR protein-specific kinase involved in mediating the interaction and localization of pre-mRNA splicing factors in mammalian cells. Journal of Cell Biology.

[48] Lai MC, Lin RI, Tarn WY (2001). Transportin-SR2 mediates nuclear import of phosphorylated SR proteins. Proceedings of the National Academy of Sciences of the United States of America.

[49] Kinoshita E, Kinoshita-Kikuta E, Takiyama K, Koike T (2006). Phosphate-binding tag, a new tool to visualize phosphorylated proteins. Molecular & Cellular Proteomics.

[50] Greaser ML (2008). Mutation that dramatically alters rat titin isoform expression and cardiomyocyte passive tension. Journal of Molecular and Cellular Cardiology.

[51] Methawasin M (2014). Experimentally increasing titin compliance in a novel mouse model attenuates the Frank-Starling mechanism but has a beneficial effect on diastole. Circulation.

[52] Warren CM, Krzesinski PR, Campbell KS, Moss RL, Greaser ML (2004). Titin isoform changes in rat myocardium during development. Mechanisms of Development.

[53] Greaser ML (2005). Developmental changes in rat cardiac titin/connectin: transitions in normal animals and in mutants with a delayed pattern of isoform transition. Journal of Muscle Research and Cell Motility.

[54] Ottenheijm CA (2009). Tuning passive mechanics through differential splicing of titin during skeletal muscle development. Biophysical Journal.

[55] Li S, Guo W, Dewey CN, Greaser ML (2013). Rbm20 regulates titin alternative splicing as a splicing repressor. Nucleic Acids Research.

[56] Beraldi R (2014). Rbm20-deficient cardiogenesis reveals early disruption of RNA processing and sarcomere remodeling establishing a developmental etiology for dilated cardiomyopathy. Human Molecular Genetics.

[57] Wyles SP (2016). Modeling structural and functional deficiencies of RBM20 familial dilated cardiomyopathy using human induced pluripotent stem cells. Human Molecular Genetics.

[58] Greaser ML, Berri M, Warren CM, Mozdziak PE (2002). Species variations in cDNA sequence and exon splicing patterns in the extensible I-band region of cardiac titin: relation to passive tension. Journal of Muscle Research and Cell Motility.

[59] Neagoe C, Opitz CA, Makarenko I, Linke WA (2003). Gigantic variety: expression patterns of titin isoforms in striated muscles and consequences for myofibrillar passive stiffness. Journal of Muscle Research and Cell Motility.

[60] Buck D (2014). Removal of immunoglobulin-like domains from titin’s spring segment alters titin splicing in mouse skeletal muscle and causes myopathy. Journal of General Physiology.

[61] Ito J (2016). RBM20 and RBM24 cooperatively promote the expression of short enh splice variants. FEBS Letters.

[62] Zhu C (2015). RBM20 is an essential factor for thyroid hormone-regulated titin isoform transition. Journal of Molecular Cell Biology.

[63] Zhu C, Yin Z, Tan B, Guo W (2017). Insulin regulates titin pre-mRNA splicing through the PI3K-Akt-mTOR kinase axis in a RBM20-dependent manner. Biochimica et Biophysica Acta.

[64] Beqqali A (2016). A mutation in the glutamate-rich region of RNA-binding motif protein 20 causes dilated cardiomyopathy through missplicing of titin and impaired Frank-Starling mechanism. Cardiovascular Research.

[65] Filippello A, Lorenzi P, Bergamo E, Romanelli MG (2013). Identification of nuclear retention domains in the RBM20 protein. FEBS Letters.

[66] Hedley ML, Amrein H, Maniatis T (1995). An amino acid sequence motif sufficient for subnuclear localization of an arginine/serine-rich splicing factor. Proceedings of the National Academy of Sciences of the United States of America.

[67] Fu XD (1995). The superfamily of arginine/serine-rich splicing factors. RNA.

[68] Graveley BR (2000). Sorting out the complexity of SR protein functions. RNA.

[69] Xiao SH, Manley JL (1997). Phosphorylation of the ASF/SF2 RS domain affects both protein-protein and protein-RNA interactions and is necessary for splicing. Genes and Development.

