# Supplementary material for: Phosphorylation of the RSRSP stretch is critical for splicing regulation by RNA-Binding Motif Protein 20 (RBM20) through nuclear localization
Source: Sci Rep. 2018 Jun 12;8:8970. doi: 10.1038/s41598-018-26624-w (PMC5997748; doi:10.1038/s41598-018-26624-w)

Supplementary Information for

Phosphorylation of the RSRSP stretch is critical for splicing regulation by RNA-Binding Motif Protein 20 (RBM20) through nuclear localization

Rie Murayama, Mariko Kimura-Asami, Marina Togo-Ohno, Yumiko Yamasaki-Kato, Taeko K. Naruse, Takeshi Yamamoto, Takeharu Hayashi, Tomohiko Ai, Katherine G. Spoonamore, Richard J. Kovacs, Matteo Vatta, Mai Iizuka, Masumi Saito, Shotaro Wani, Yuichi Hiraoka, Akinori Kimura* and Hidehito Kuroyanagi*

*To whom correspondence should be addressed.
Hidehito Kuroyanagi, PhD, Phone: +81(Japan)-3-5803-4695; E-mail: kuroyana.end@tmd.ac.jp, or
Akinori Kimura, MD, PhD, Phone: +81(Japan)-3-5803-4905; E-mail: akitis@mri.tmd.ac.jp

This file includes

- Supplementary Table 1. Sequences of primers used in plasmid construction.
- Supplementary Table 2. Sequences of primers used to amplify alternatively spliced mRNAs from the endogenous genes or the splicing reporter minigenes in the RT-PCR assays.
- Full-length gels and blots for Figures 1C, 2C, 2D, 3D, 4A-C, 5B-D and 6B-C.

Supplementary Table 1. Sequences of primers used in plasmid construction.

Primers used to amplify genomic fragments.

Amplified genomic fragments	Sequence
Mouse *Ttn* exon 50 – exon 51	5′-CACCATGGCCCCTGTGATCAAAAGGAGAATTG-3′
	5′-AGTGGTGCAGGCTCCACTTTTCTGACAGCGACCGCAGC-3′
Mouse *Ttn* exon 218 – exon 219	5′-GGAGCCTGCACCACTAaaAAAGGTATCTCACAGGCTG-3′
	5′-CCGCTTTTCgGCACCACCTCTTCCTTTGGC-3′
Human histone H2B	5′-TGGCTTATCGAAATTCCGCCACCATGCCAGAGCCAGCGAAGTCT-3′
	5′-ACAAACTTGTGCCCTTCTTAGCGCTGGTGTACTTG-3′

Underlines indicate adaptor sequences for directional TOPO or In-Fusion cloning. Lower case indicates mutations for a frameshift or disruption of a nonsense codon.

Primers used to amplify cDNA fragments.

Amplified cDNA fragments	Sequence
Mouse full-length RBM20	5′-CACCATGGTGCTGGCAGTAGCCATGAG-3′
	5′-TCATAGCTTCTTCCTTTCCA-3′
Mouse RBM20(517-657)	5′-CAGGCTCCACCATGGGCGCTGGACGGGTAGTGCACA-3′
	5′-AAGAAAGCTGGGTCTACCGAGAGGGGCCTGGGGGACTAT-3′

Underlines indicate adaptor sequences for directional TOPO or In-Fusion cloning.

Primers used for nucleotide mutagenesis.

Mutation sites	Sequence
Human H2B cryptic splice site	5′-AGCGCATCGCAGGaGAGGCTTCCCG-3′
	5′-CGGGAAGCCTCtCCTGCGATGCGCT-3′
Mouse RBM20^{S637A}	5′-GCGGCCACGTgCTCGAAGTCCAATGAGCCG-3′
	5′-TGGACTTCGAGcACGTGGCCGCTCTGGACC-3′
Mouse RBM20^{S639A}	5′-CACGTTCTCGAgcTCCAATGAGCCGATCAC-3′
	5′-GCTCATTGGAgcTCGAGAACGTGGCCGCTC-3′
Mouse RBM20$^{S637A/S639A}$	5′-GCGGCCACGTgCTCGAgcTCCAATGAGCCGATCAC-3′
	5′-GGCTCATTGGAgcTCGAGcACGTGGCCGCTCTGGAC-3′
Mouse RBM20^{R636W}	5′-CCAGAGCGGCCAtGgTCTCGAAGTCCAATGAGC-3′
	5′-GCTCATTGGACTTCGAGAcCaTGGCCGCTCTGG-3′
Mouse RBM20^{D868N}	5′-AAGGGACAAaACTGTGACAGTGGGAGT-3′
	5′-CTCCCACTGTCACAGTtTTGTCCCTT-3′
Mouse RBM20^{G1009X}	5′-AtGAGAGGAGGACTCAGATGTGA-3′
	5′-ATCTGAGTCCTCCTCTCaTCTTGCCTCCTTC-3′
Mouse RBM20^{P1059R}	5′-CAGCCgCCTGGAAGGAAAAG-3′
	5′-CCTTCCAGGcGGCTGGCTTC-3′
Mouse RBM20^{E1178K}	5′-GCTGTCTGTCCCCTtCGTCTCCTTC-3′
	5′-GAGACGaAGGGGACAGACAGCC-3′

Lower case indicates nucleotides for mutation.

Supplementary Table 1. (continued)

Primers used for deleting conserved domains from RBM20 cDNA.

Constructed RBM20 mutants	Sequence
Mouse RBM20$^{\Delta 54-149}$	5′-GCCTCAGCCTCAA GTGTCCCAGCATG-3′
	5′-CATGCTGGGACAC TTGAGGCTGAGGC-3′
Mouse RBM20$^{\Delta 395-441}$	5′-TGCTGTCCGTGAGGCCC CTGCTCTTCTCAGAAAGTGCTGG-3′
	5′-CCAGCACTTTCTGAGAAGAGCAG GGGCCTCACGGACAGCA-3′
Mouse RBM20$^{\Delta 519-600}$	5′-GAGGAAAGGCGCT CTGAAGAAACCTGGGAAAAATGTGG-3′
	5′-CCACATTTTTCCCAGGTTTCTTCAG AGCGCCTTTCCTC-3′
Mouse RBM20$^{\Delta 825-924}$	5′-GACCAAGAAGGAGCT TGTACCTGTGTAACGG-3′
	5′-AGCTCCTTCTTGGTC-3′
Mouse RBM20$^{\Delta 1106-1172}$	5′-AGCTGAAAGAGCCCCTT GGCCTGAAGGAGACGG-3′
	5′-CCGTCTCCTTCAGGCC AAGGGGCTCTTTCAGCT-3′

Gaps indicate boundaries between upstream and downstream fragments.

Primers used to amplify vector backbones.

Vector backbones	Sequence
pDEST-cDNA3	5′-AATTTCGATAAGCCAGTAAGCA-3′
	5′-AGGGCACAAGTTTGTACAAAAAA-3′
pDEST-cDNA3-FLAG-3xNLS	5′-GATCCAAAAAAGAAGAGAAAGGTAGATCCAAAAAAGAAGAGAAAGGTAG ATCCAAAAAAGAAGAGAAAGGTAAACACAAGTTTGTACAAAAAAGCTG-3′
	5′-CTTCTTTTTTGGATCCTTGTCATCGTCGTCCTTGTAGTCCAT-3′
pcDNA3-FLAG	5′-GTACAAACTTGTGTTCTTGTCATCGTCG-3′
	5′-GTACAAAGTGGTCCTAGAGCTCGCTGATCAG-3′

Underlines indicate sequences encoding NLSs or *attB* sequences for homologous recombination.

Supplementary Table 2. Sequences of primers used to amplify alternatively spliced mRNAs from the endogenous genes or the splicing reporter minigenes in the RT-PCR assays.

Species	Gene	Sequence	Position & Direction
Reporter	*Ttn*	5’-GAGATCCAAGGAGCCCCCAA-3’	Exon 50, Forward
		5’-TGTGGCCGTTTACGTCG-3’	EGFP, Reverse
		5’-CTCGATCTCGAACTCGTG-3’	mCherry, Reverse
Mouse	*Camk2d*	5′-AAGGGCGCCATCTTGACAAC-3′	Exon 12-13, Forward
		5′-TCAAAGTCCCCATTGTTGAT-3′	Exon 19, Reverse
	Ldb3	5′-TCCAAGCGGCCTATTCCCATC-3′	Exon 4, Forward
		5′-TGTATTCTGTCCCGGTCATCTG-3′	Exon 9, Reverse
	Rbm20	5′-ACATGCTCCGGGAAGCTGACAG-3′	Exon 8, Forward
		5′-GGATAGTGTTTCCGGTCATGTG-3′	Exon 9, Reverse
	Ttn	5’-GAGATCCAAGGAGCCCCCAA-3’	Exon 50, Forward
		5’-TCTGCCAGATGATCTCAATCAC-3’	Exon 51, Reverse
		5’-CACAGAGCCATATGAAGAACCCTA-3’	Exon 115, Forward
		5’-AAAGCAACCATTACCATTGGTAGA-3’	Exon 215, Forward
		5’-GCACCACCTCTTCCTTTGGC-3’	Exon 219, Reverse

Assay Class: DNA 7500 Created: 04/15/2018 21:33:25
Data Path: E:\...-16\2100 expert_DNA 7500_DE72903049_2018-04-16_13-33-25.xad Modified: 04/16/2018 16:41:48

Gel Image

Figure 1C

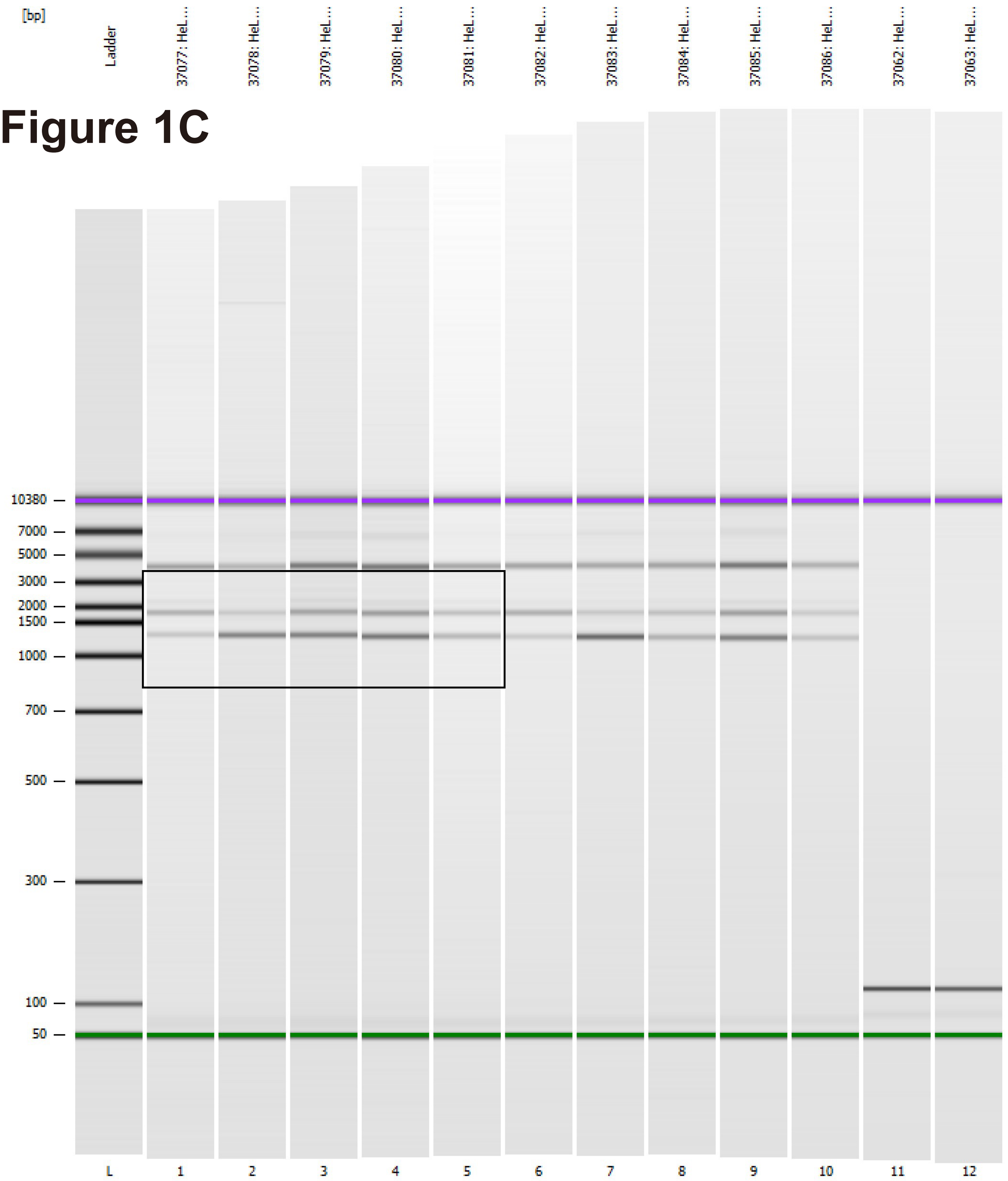

 © Copyright 2003 - 2009 Agilent Technologies, Inc.

Assay Class: DNA 7500
Data Path: \\1...-22\2100 expert_DNA 7500_DE72903049_2013-11-22_17-00-44.xad

Created: 2013/11/22 17:00:44
Modified: 2013/11/22 19:31:09

Gel Image

Figure 2C

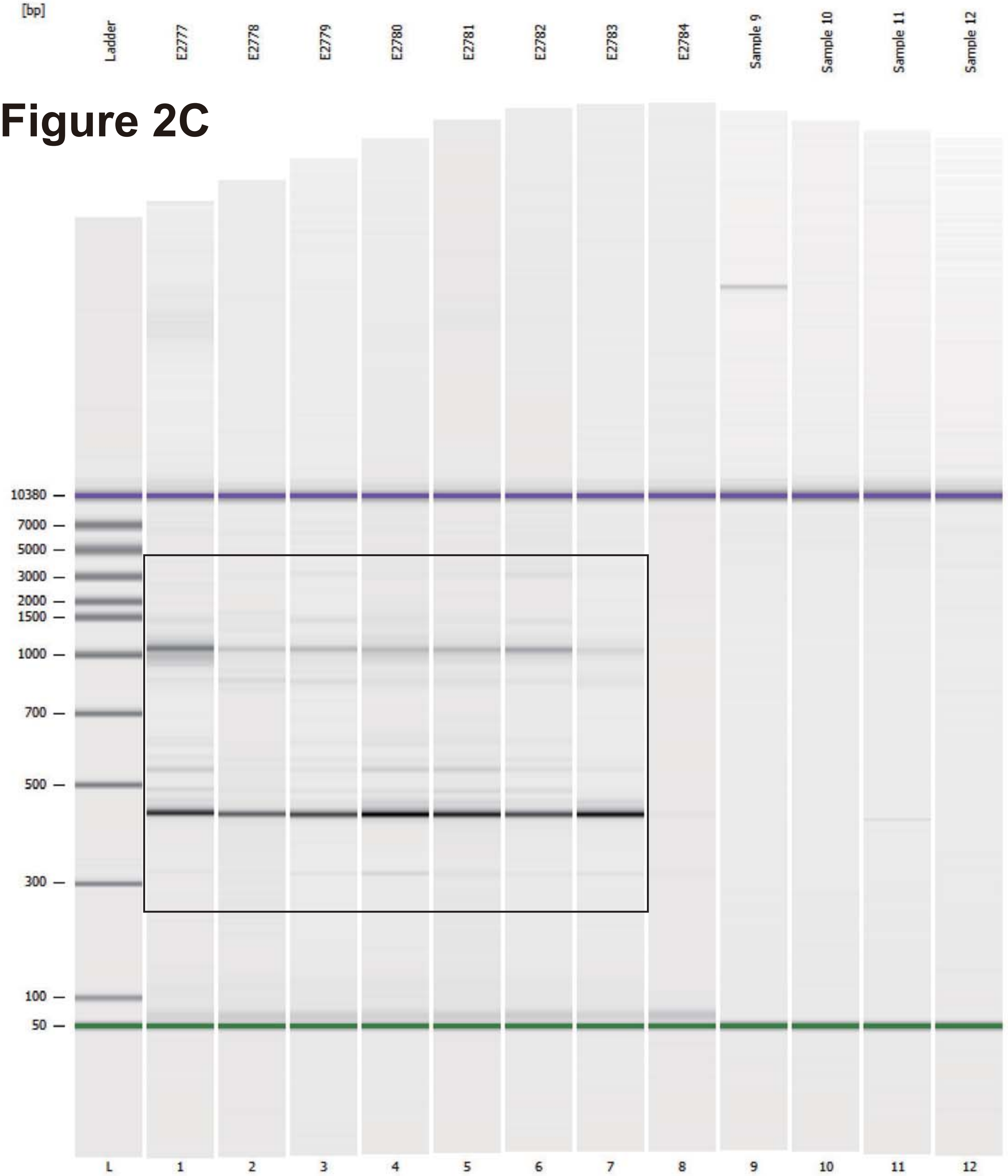

 © Copyright 2003 - 2009 Agilent Technologies, Inc.

Assay Class: DNA 7500 Created: 03/28/2018 19:43:19
Data Path: E:\...-29\2100 expert_DNA 7500_DE72903049_2018-03-29_11-43-19.xad Modified: 04/02/2018 18:31:18

Gel Image

Figure 2D

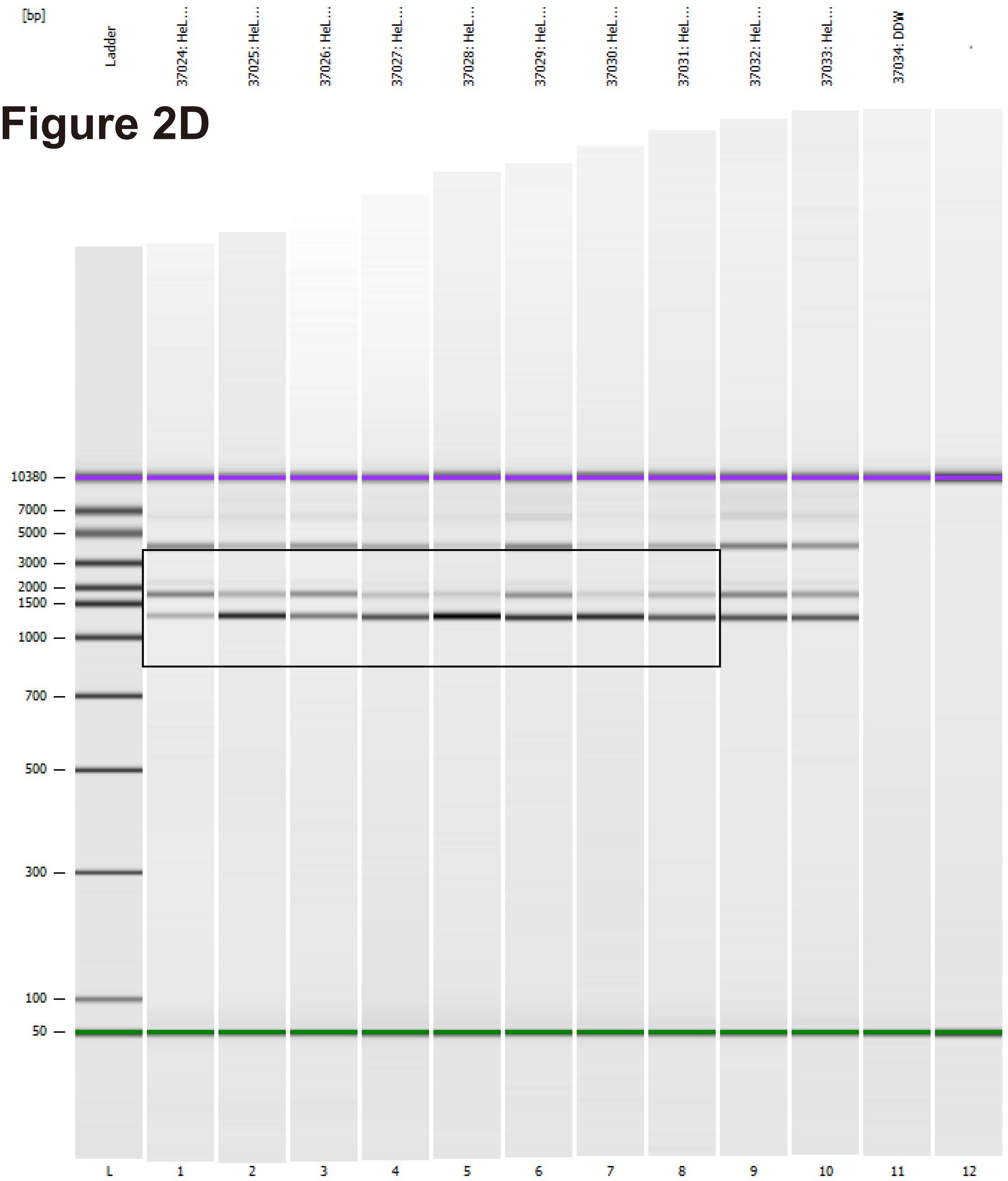

 © Copyright 2003 - 2009 Agilent Technologies, Inc.

Assay Class: DNA 7500
Data Path: \\1...-06\2100 expert_DNA 7500_DE72903049_2015-01-06_19-22-57.xad
Created: 2015/01/06 19:22:57
Modified: 2015/01/06 20:37:54

Gel Image

Figure 3D

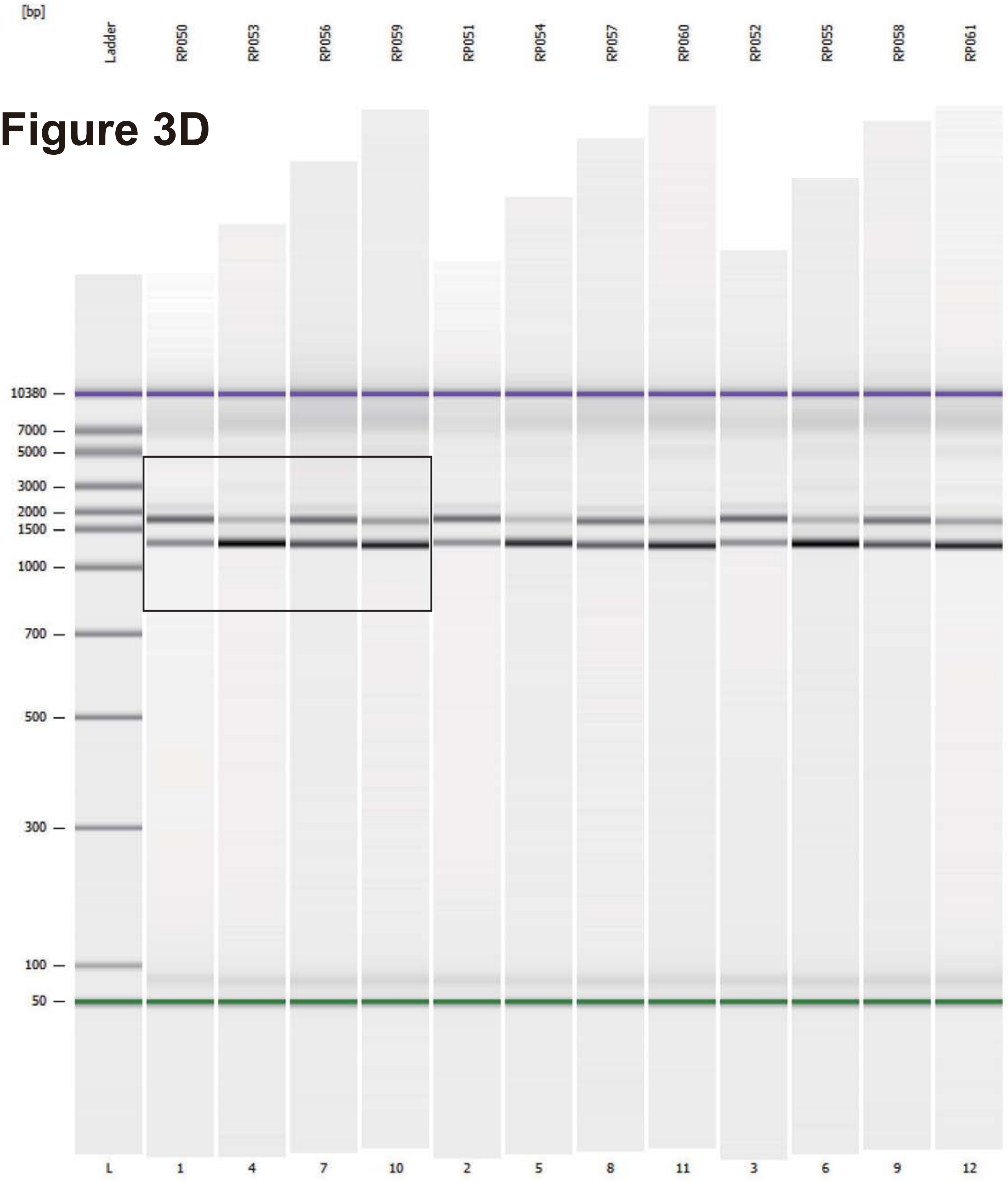

 © Copyright 2003 - 2009 Agilent Technologies, Inc.

Figure 4A

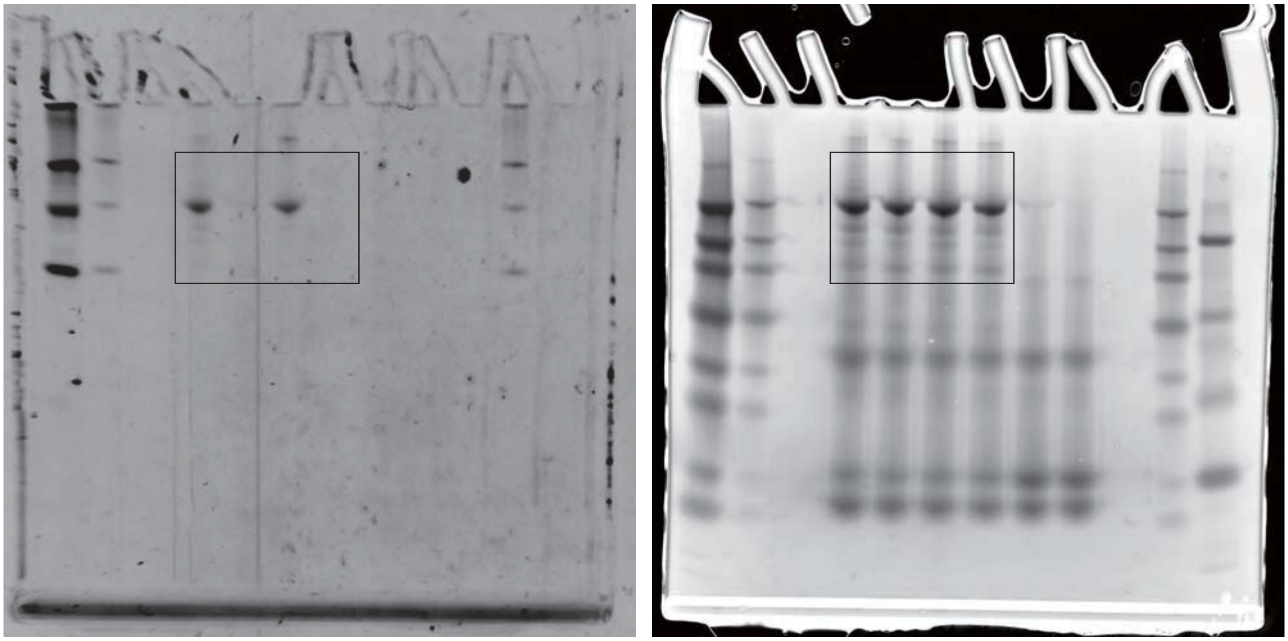

Figure 4B

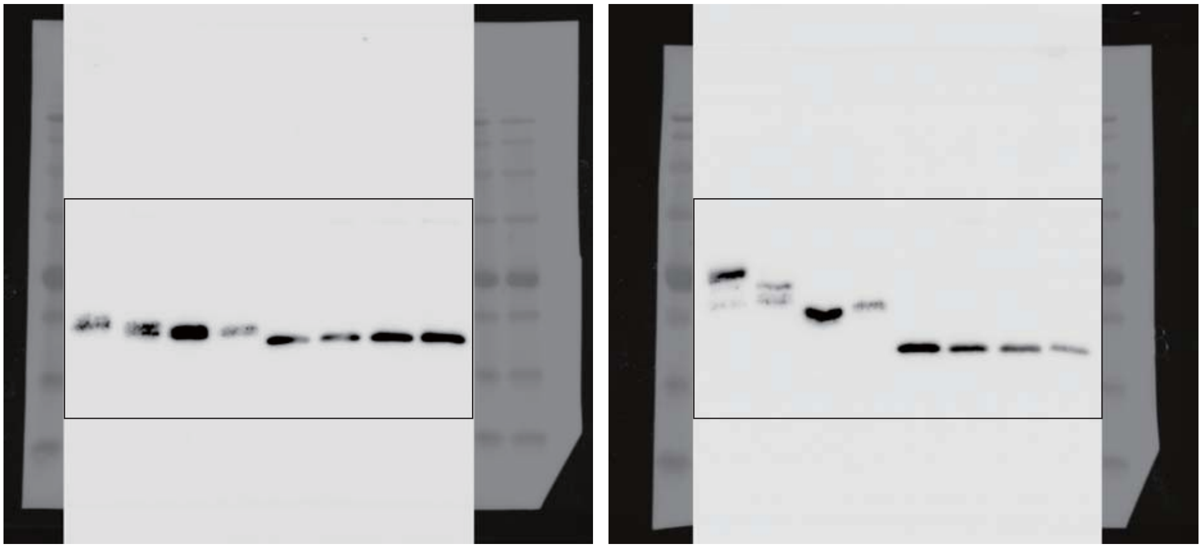

Figure 4C

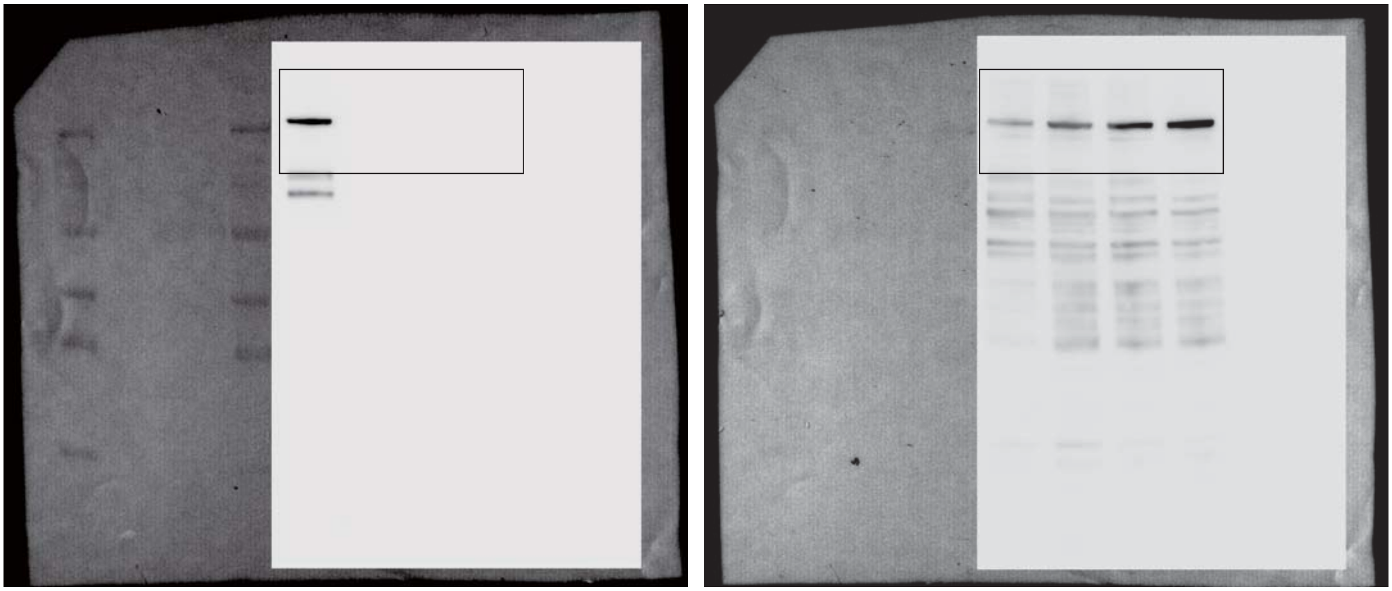

Figure 5B

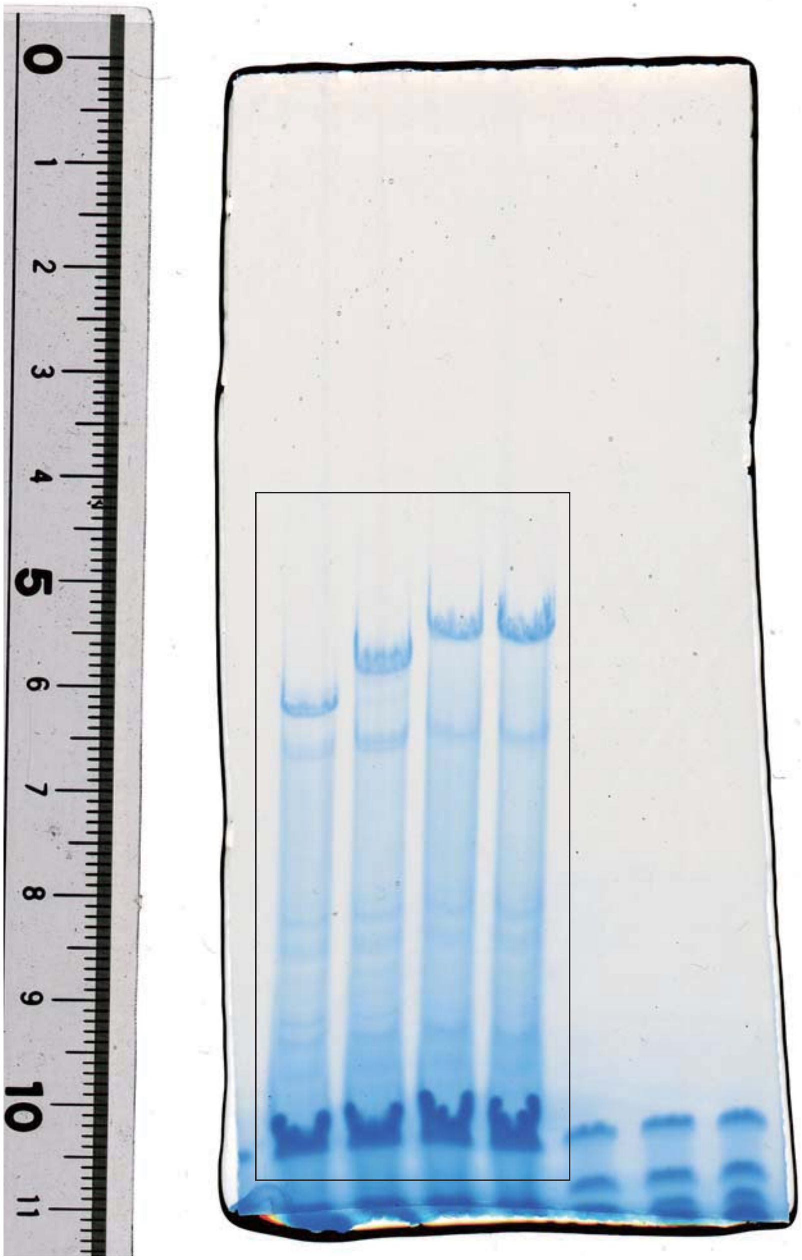

Assay Class: DNA 1000
Data Path: \\1...agi\2100 expert_DNA 1000_DE72903049_2017-11-16_14-46-20.xad

Created: 2017/11/16 14:46:19
Modified: 2017/11/17 10:10:04

Gel Image

Figure 5C

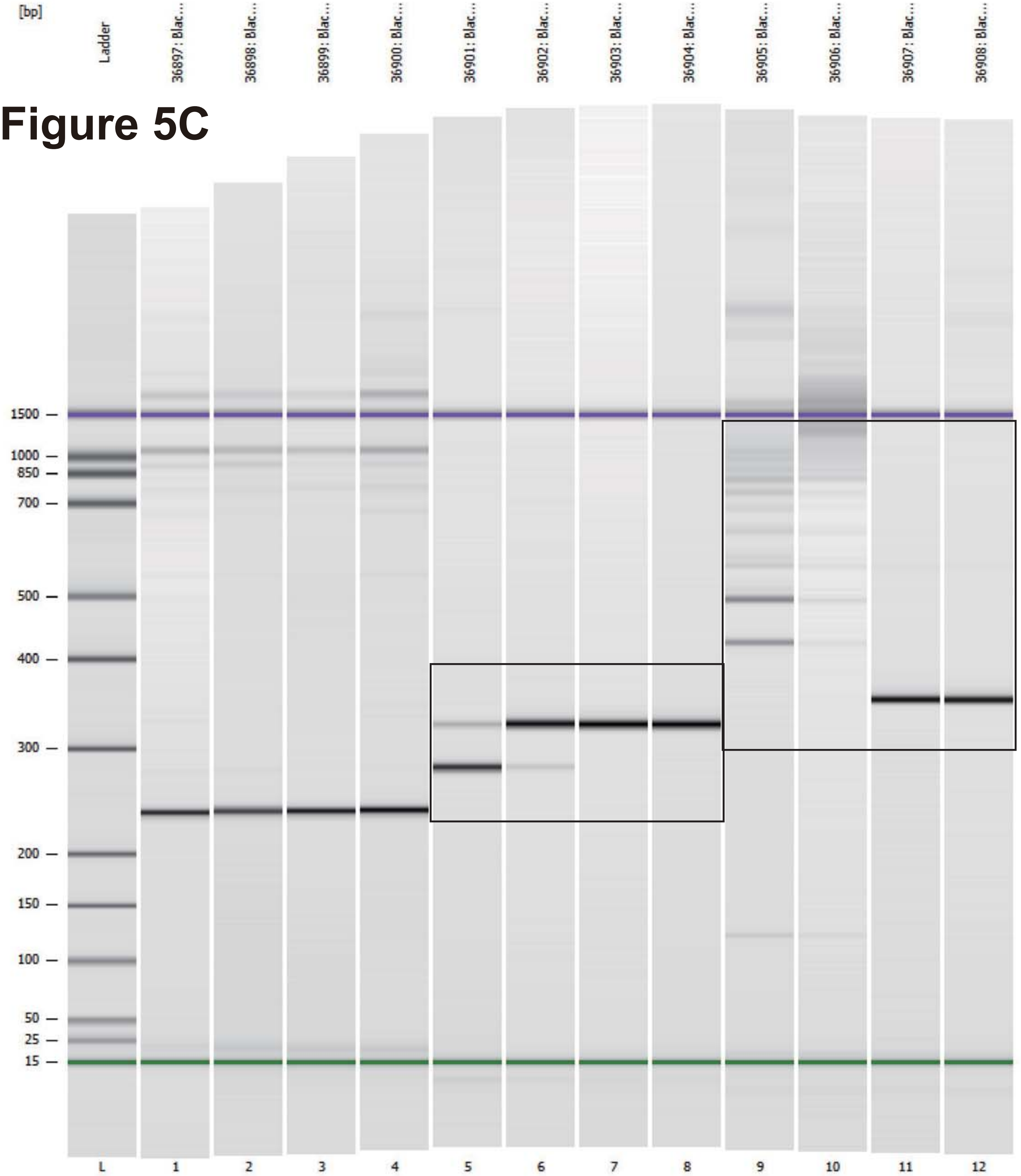

 © Copyright 2003 - 2009 Agilent Technologies, Inc.

Assay Class: DNA 1000
Data Path: \\1...agi\2100 expert_DNA 1000_DE72903049_2017-11-16_15-49-25.xad

Created: 2017/11/16 15:49:24
Modified: 2017/11/17 11:04:35

Gel Image

Figure 5C, 5D

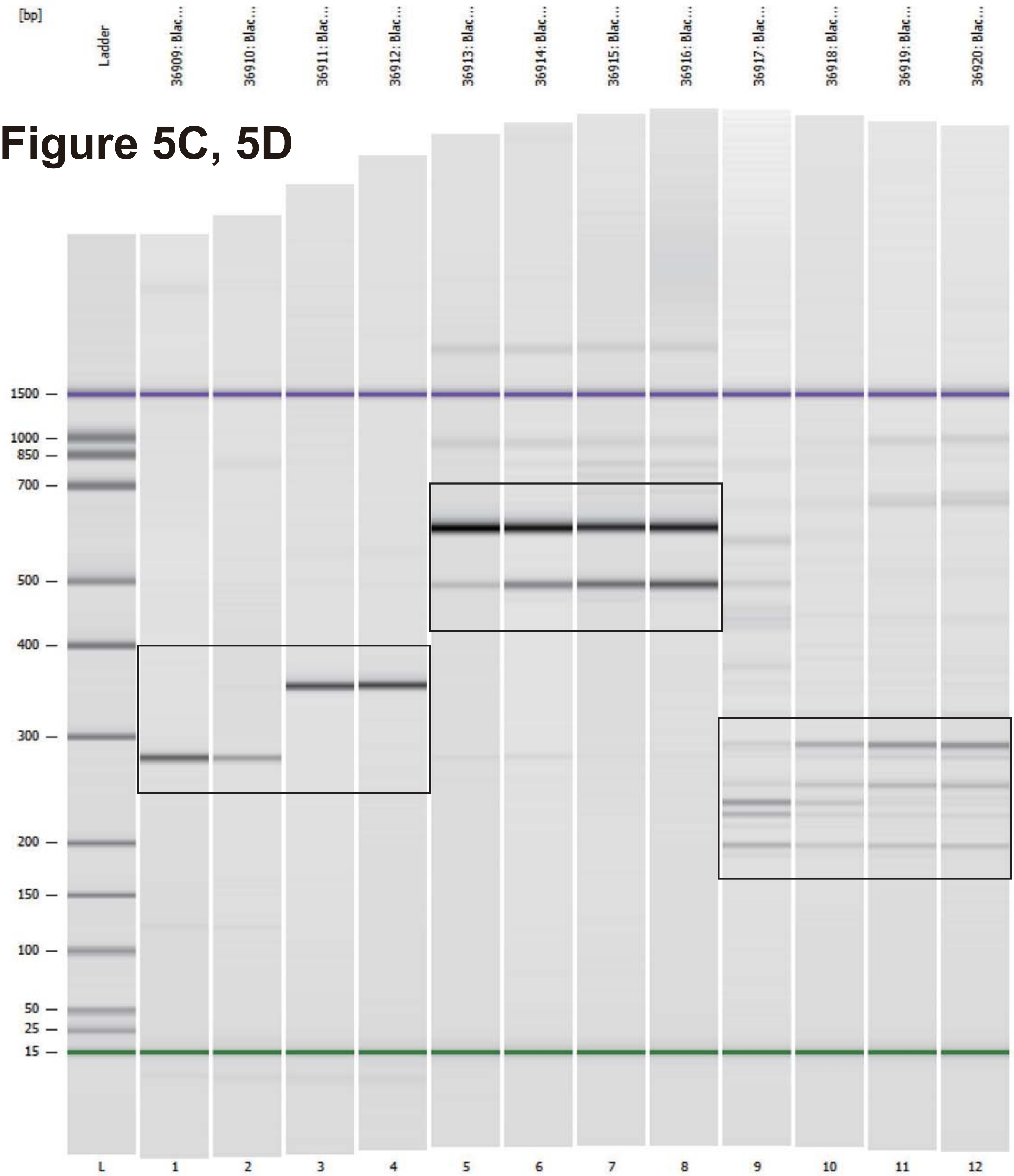

 © Copyright 2003 - 2009 Agilent Technologies, Inc. Printed: 2017/12/22 19:40:42

Assay Class: DNA 7500
Data Path: \\1...agi\2100 expert_DNA 7500_DE72903049_2016-01-28_13-29-07.xad
Created: 2016/01/28 13:29:06
Modified: 2016/01/30 19:25:30

Gel Image

Figure 6B

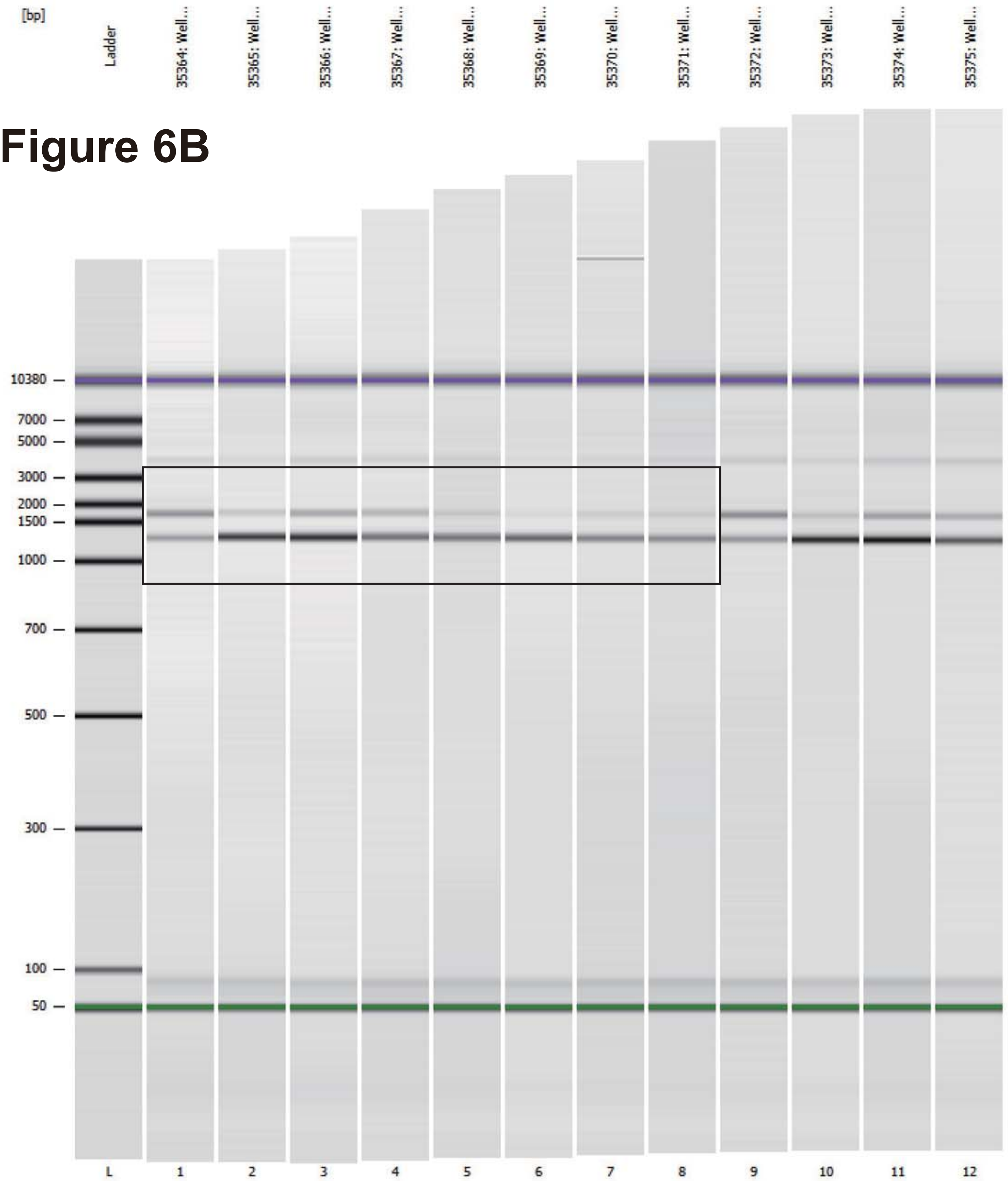

 © Copyright 2003 - 2009 Agilent Technologies, Inc.

Figure 6C

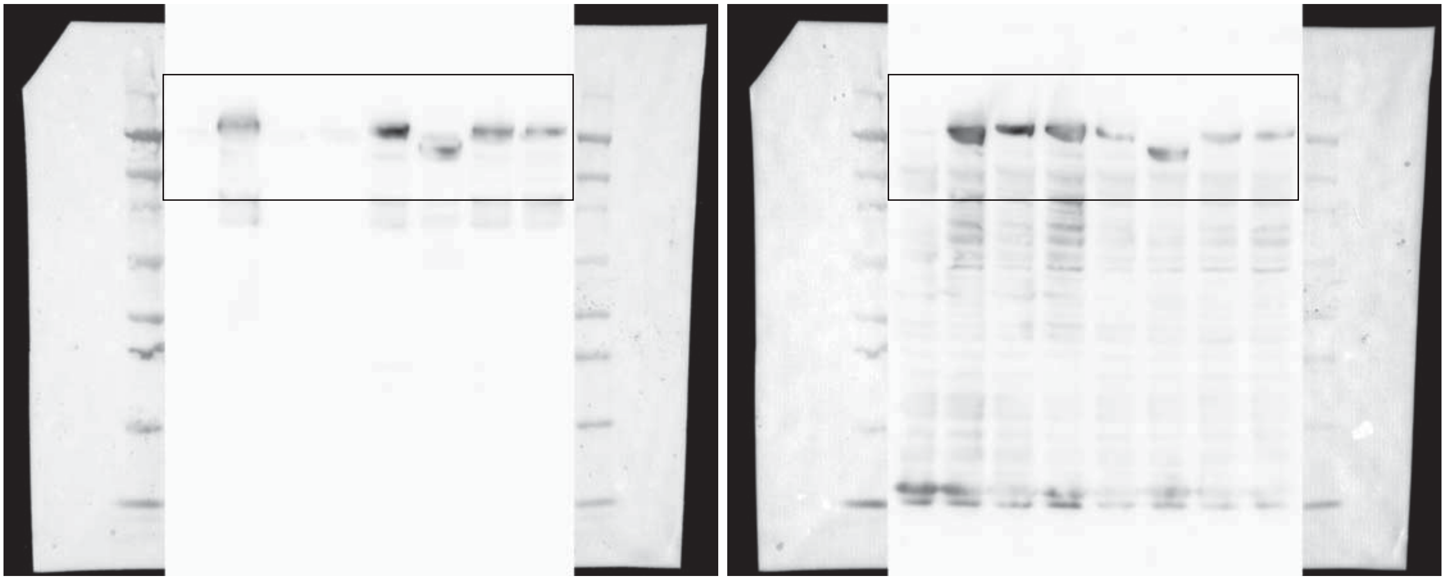

Supplement: Supplementary file 1 — Supplementary Information [file 41598_2018_26624_MOESM1_ESM.pdf]
